# Exercise Training for Mild Cognitive Impairment Adults Older Than 60: A Systematic Review and Meta-Analysis

**DOI:** 10.3233/JAD-220243

**Published:** 2022-08-16

**Authors:** Hui Li, Wenlong Su, Hui Dang, Kaiyue Han, Haitao Lu, Shouwei Yue, Hao Zhang

**Affiliations:** a Rehabilitation Center, Qilu Hospital, Cheeloo College of Medicine, Shandong University, Jinan, Shandong, China; b China Rehabilitation Research Center, Beijing, China; c University of Health and Rehabilitation Sciences, Qingdao, Shandong, China

**Keywords:** Aged, exercise, meta-analysis, mild cognitive impairment, systematic review

## Abstract

**Background:**

The prevalence of mild cognitive impairment (MCI) continues to increase due to population aging. Exercise has been a supporting health strategy that may elicit beneficial effects on cognitive function and prevent dementia.

**Objective:**

This study aimed to examine the effects of aerobic, resistance, and multimodal exercise training on cognition in adults aged > 60 years with MCI.

**Methods:**

We searched the Cochrane Library, PubMed, and Embase databases and ClinicalTrials.gov (https://clinicaltrials.gov) up to November 2021, with no language restrictions. We included all published randomized controlled trials (RCTs) comparing the effect of exercise programs on cognitive function with any other active intervention or no intervention in participants with MCI aged > 60 years.

**Results:**

Twelve RCTs were included in this review. Meta-analysis results revealed significant improvements in resistance training on measures of executive function (*p* < 0.05) and attention (*p* < 0.05); no significant differences were observed between aerobic exercise and controls on any of the cognitive comparisons.

**Conclusion:**

Exercise training had a small beneficial effect on executive function and attention in older adults with MCI. Larger studies are required to examine the effects of exercise and the possible moderators.

## INTRODUCTION

Alzheimer’s disease (AD) is a neurodegenerative disorder that is the most common cause of dementia [1]. In the initial stage of AD, the decline in cognitive function can be significantly subtle and is currently being clinically identified as degenerative mild cognitive impairment (MCI) [2]. There are three clinical subtypes of MCI: amnestic MCI, MCI involving various degrees of impairment in multiple cognitive domains, and MCI involving a single non-memory domain. Patients with MCI normally have self- or informant-reported cognitive complaints [3] and documented objective impairment in one or more cognitive domains that is greater than would be expected for the patient’s age or educational background [4], but still preserved the activities of daily living without significant impairment in social or occupational functions [5]. The prevalence of MCI increases with age (6.7% for ages 60–64 years, 65.2% in older adults aged > 60 years), and patients with MCI are at a higher risk of progressing to dementia than age-matched controls (progression to dementia at a rate of approximately 14.9% in MCI aged > 65 years followed by 2 years) [6]. To date, there is no pharmacologic management currently approved for MCI, and behavioral training, such as cognitive therapy and exercise training, has symptomatic cognitive benefits in MCI [7–9].

Considering the feasibility and acceptability of intervention training, exercise seems to be an available and economical therapeutic intervention for individuals at an earlier point of cognitive decline. Evidence indicates that exercise elicits cognition- and neurovascular oxygenation-promoting effects in rodents by reducing amyloid pathology [10–12]; enhancing hippocampal neurogenesis [13], oxidative stress [14–16], and intracranial energy metabolism [17]; and reducing neuroinflammation [18]. However, findings are mixed in human studies. Evidence suggests that exercise reduces vascular risk factors, such as hypertension [19], dyslipidemia [20], type 2 diabetes [21], obesity [22], subclinical atherosclerosis [23], and arrhythmias [24], which are associated with a greater risk of cognitive impairment and dementia [25]. Moreover, exercise may reduces the risk of stroke [26]. Recent meta-analyses showed that physical exercise may help preserve or even improve cognitive function [27–29] and peripheral brain-derived neurotrophic factor concentrations [30, 31] in healthy older adults. Exercise increases precentral cortical thickness and reduces neuroplasticity [32]; augments gray and white matter volumes [33], hippocampal volume [34–36], and systemic levels of glycosylphosphatidylinositol-specific phospholipase; and ameliorates age-related regenerative impairments [37].

Previous studies have reported a valid effect of exercise training on cognitive function in older adults with and without cognitive impairment [38–40] with a general effect of exercise training [41–43]. For instance, all levels of physical activity were reported to have a significant and consistent protective effects against the occurrence of cognitive decline [44]. Nevertheless, studies on the effects of physical exercise in older adults with MCI are insufficient and vary in efficacy owing to the large variability in exercise protocols, compliance, and complicated interpretation of the results [43]. This review involved patients with MCI aged > 60 years, when the global prevalence rates of dementia are exacerbated continuously. We compared the results with previous reviews by examining the effects of aerobic, resistance, mind–body, and multimodal exercise training on cognitive performance in patients with MCI. These results may be helpful in clarifying the efficacy of exercise interventions on cognitive function in older adults with MCI.

## MATERIALS AND METHODS

This study was conducted according to the Preferred Reporting Items for Systematic Reviews and Meta-Analysis. The study protocol was registered with the International Prospective Register of Systematic Reviews (CRD42021259555).

### Search strategy

A librarian-led systematic search of Embase, PubMed, and Cochrane databases was performed using a strategy combining selected Medical Subject Headings terms (exercise, exercise therapy, resistance training, sports, physical fitness, circuit-based exercise, endurance training, Tai Ji, bicycling, yoga, walking, jogging, running, swimming, muscle stretching exercises, plyometric exercise, cognitive dysfunction, aged) and free-text terms (For full search strategy, see the Supplementary Material). No language or other limitations were imposed during the search process. Additionally, we searched ClinicalTrial.gov, reference lists of selected articles, and related review articles to screen for related clinical trials being conducted. The search terms regarding random control study design (randomized controlled trial [Publication Type] OR randomized [Title/Abstract] OR placebo [Title/Abstract]) were obtained from McMaster University Health Information’s website. The last search was performed on November 5, 2021.

### Criteria for considering studies for this review

#### Types of studies

Human subject studies designed as randomized controlled trial (RCT) published in English were included without year restriction. Nonrandomized controlled studies, conference abstracts, reviews, protocols, and secondary analyses were excluded.

#### Types of participants

Participants diagnosed with MCI according to accepted criteria (Petersen, Winblad, Morris, Albert criteria) and aged > 60 years were included. We excluded healthy aging elderly adults, patients with any form of dementia, or those diagnosed with cognitive impairment due to definite etiologies, such as trauma or vascular or psychiatric diseases. Any clinical subtype of MCI was considered eligible. MCI defined by a single scale was considered insufficiently credible and unreliable.

#### Types of interventions

We included supervised structured exercises of any frequency, intensity, duration, or time directed at improving physical fitness. Combined exercise interventions with two or more types were included as multimodal training; however, studies involving exercise programs that combined cognitive training or other non-exercise activities (e.g., diets, drugs, and video games), not fully supervised or lasted < 4 weeks, were excluded.

#### Types of control groups

We included studies that involved comparators who engaged in a strength or balance tone, a program of social or mental activities, no treatment, or on the waiting list.

#### Types of outcomes

**Fig. 1:**
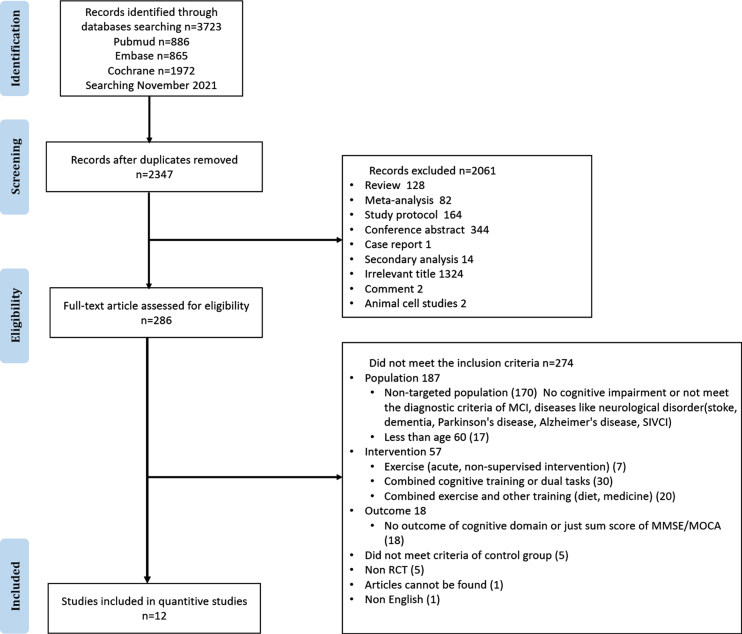
PRISMA flow diagram of the literature selection process.

The primary outcome was cognitive function that was evaluated using a measurable cognitive screening instrument, whereas the secondary outcome was the dropout rate. Studies with no outcomes in specific domains of cognitive fields or studies that only involved the Mini-Mental State Examination (MMSE) or Montreal Cognitive Assessment (MoCA) were excluded. Two authors (WS and KH) initially excluded articles that did not meet the inclusion criteria, based on their titles and abstracts. After deduplication, the remaining articles were independently screened for eligibility by full-text assessment based on the defined inclusion criteria. Disagreements were resolved through discussions with expert HZ (study selection procedure, Fig. 1).

### Quality assessment and data extraction

**Fig. 2:**
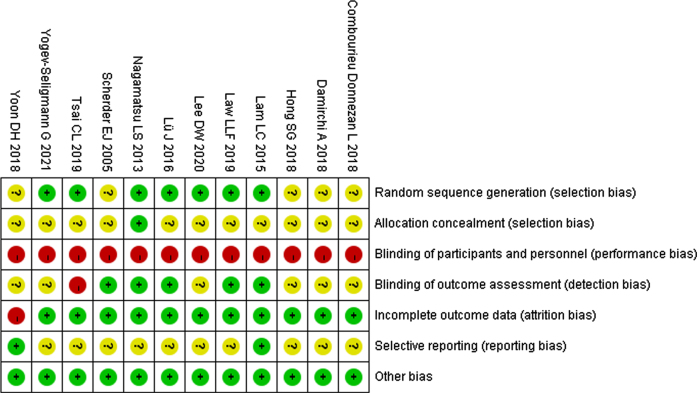
Cochrane’s Quality Assessment: summary of risk of bias for each quality item for each included study.

We extracted types of data from the full-text articles, including study characteristics of author; published year; country; RCT design, including grouping, sample size, sex ratio, mean age, MMSE score before the intervention, and diagnostic criteria; characteristics of the exercise intervention group, including type, intensity, frequency, duration, and time; and outcome neuropsychological tool used for measuring cognitive function. If the full text or data were incomplete, the author was contacted via email. The protocol of each included study was examined to verify reporting bias. The quality of individual study was assessed using the Cochrane Risk of Bias Tool (Fig. 2) [45]. Risk was assessed to be “low,” “high,” or “unclear.” Two independent authors (HL and HD) assessed the risk of bias. Disagreements were resolved by discussion with a third author.

### Data synthesis and statistical analyses

Review Manager (RevMan) version 5.3 was used for all analyses. The summary statistics required for each assessment for continuous data were the post-intervention mean scores, standard deviation, and number of participants in each group (several studies did not provide change scores). The mean was entered as negative when a higher score indicated worse performance (e.g., reaction time, trail making test). Cognitive function is normally assessed using both global and individual cognitive tests. There are general cognitive tests (MMSE, MoCA) and specific tests that cover cognitive domains, including memory, attention, visuospatial processing, language, executive function, and social comportment. We classified the cognitive outcomes assessed using various neuropsychological tests into seven domains: (1) immediate memory, (2) working memory, (3) delayed memory, (4) processing speed, (5) attention, (6) executive function, and (7) recognition [27]. For each domain, we included only one test from a single trial that was used more frequently or closer to the domain. For dichotomous data, we extracted the number of participants in each group for each trial. Statistical analyses were performed if more than one study could be grouped into cognitive domains according to intervention (e.g., aerobic or resistance exercise). When only one study involved a category, the statistics from that single study were reported, but not analyzed. For continuous variables measured on different scales, standardized mean differences (SMDs) and 95% confidence interval (CI) were calculated; for dichotomous variables, risk ratios and 95% CIs were calculated.

The I^2^ statistic was calculated to describe the proportion of true heterogeneity in the observed variance across studies. Cochrane’s handbook recommends heterogeneity as not important (0–40%), moderate heterogeneity (30–60%), substantial heterogeneity (50–90%), and considerable heterogeneity (75–100%). Sensitivity analysis was performed when I^2^ was > 50% [46]. For which data were available, meta-analyses were performed using the inverse-variance method. A random-effects model was used to assess the cognitive outcomes and dropout rates. Given the small number of included studies, we did not perform a funnel plot visual test to investigate publication bias [9, 47].

## RESULTS

### Included studies

The initial database search identified 3,723 records (PubMed, *n* = 886; Embase, *n* = 865; Cochrane, *n* = 1,972) (Fig. 1). After duplicates were removed and screened, the full texts of 286 articles were assessed for eligibility, of which 12 met the eligibility criteria (Tables 1–3). A total of 708 participants were included and assessed in this systematic review. Seven trials of 122 participants assessed the treatment effects of aerobic exercise, including bicycle or motor-driven treadmill training [48–51] (*n* = 66) and walking [52–54] (*n* = 56); six trials of 116 participants reported on resistance exercise, including elastic band training [55–57] (*n* = 48), circuit exercise [51, 53] (*n* = 46), and dumbbell training group [58] (*n* = 22). Moreover, one trial researched on multimodal exercise [59] (*n* = 147). In total, 112 participants with MCI in the control groups were compared to the aerobic training group, who received a waiting list, a maintaining lifestyle routine, a strength or balance tone, muscle-stretching exercises, or social visits; 126 participants in the control groups were compared to the resistance training group, who received a regular lifestyle routine, a strength or balance tone, stretching exercises, or lecture counseling classes; and 131 participants in the control groups were compared with the multimodal exercise group. Three mind–body training studies were excluded because their exercise protocol involved home-based exercise and lacked supervision [60–62]. No further articles were found by searching the reference lists of reviews identified during the initial search or in the included articles.

**Table 1:** Characteristics of studies –aerobic exercise versus control group

Study	RCT design	Country	Group (N)	Sex (male/female)	Mean age	MMSE	Diagnosis criteria	Exercise	Outcome
Tsai et al. [51]	Parallel	China	Aerobic exercise (AE) *n* = 19 Resistance exercise (RE) *n* = 18 Static stretching exercises (CO) *n* = 18	5/14 (AE) 7/11 (RE) 5/13 (CO)	66.00 (AE) 65.44 (RE) 65.17 (CO)	27.16±1.26 (AE) 26.56±1.34 (RE) 27.00±1.65 (CO)	Gauthier 2006, Petersen 2004, Winblad 2004	AE: Type: Bicycle ergometer or motor-driven treadmill Intensity: 70–75% of the target heart rate reserve (HRR) Frequency: 3 sessions/week Duration: 40 min Time: 16 weeks CO: Type: Static stretching exercises Intensity: No load Frequency, duration, and time: same to AE group	**Working memory**: Digit span of Wechsler-IV Adult intelligence test
Yogev-Seligmann et al. [50]	Parallel	Israel	AE *n* = 13 Balance and tone (BAT) *n* = 14	8/5 (AE) 7/7 (BAT)	70.84 (AE) 71.92 (BAT)	Not mentioned	Albert 2011	AE: Type: Stationary bicycles Intensity: 70% to 80% HRR; Frequency: 3 sessions/week Duration: 40 min Time: 16 weeks BAT: Type: balance, gross motor coordination, and light toning exercises Intensity: maintaining heart rate below 30% of HRR Frequency, duration, and time: same to AE group	**Immediate and delayed memory**: The Rey Auditory Verbal Learning Test **Working memory**: Digit span component of the Wechsler memory test **Processing speed**: The color version of the trail making test 1 **Execution function**: The verbal fluency test **Attention**: The color version of the trail making test 2 **Recognition**: Faces/houses recognition test
Scherder et al. [52]	Parallel	America	AE *n* = 15 Hand and face exercises Control (CO) *n* = 15	2/13 (AE) 1/14 (CO)	84 (AE) 86 (CO)	9.73±1.94 (AE) 9.87±1.41 (CO)	Petersen 1999	AE: Type: Self-paced slow walking with an aid Frequency: 3 sessions/week Duration:30 mins Time:6 weeks CO: One subgroup eight subjects received social visits as a ‘treatment’, while in the other subgroup the remaining seven subjects continued their normal social activities.	**Executive function**: Retrieve familiar information from semantic memory (Category Naming); **Processing speed:** Trail-making A + B **Working memory**: Digit span from the Wechsler Memory Scale— Revised **Memory:** Visual Memory Span, The Verbal Learning and Memory Test, Direct Recall; Rivermead Behavioural Memory Test (faces, pictures) **Delayed memory and recognition**: The Verbal Learning and Memory Test, delayed recall and recognition.
Nagamatsu et al. [53]	Parallel	Canada	AE *n* = 30 RE *n* = 28 BAT *n* = 28	27.4±1.5 (AE) 27.0±1.8 (RE) 27.1±1.7 (CO)	All female	75.6 (AE) 73.9 (RE) 75.1 (CO)	Petersen2004	AE: Type: Outdoor walking Intensity: 70% to 80% of HRR, 13–15 on the RPE scale Frequency: 2 sessions/week Duration: 60 min Time: 26 weeks BAT: Type: Stretching exercises, range of motion exercises, balance exercises functional and relaxation techniques Intensity: No loading Duration and time: same with RE group	**Verbal memory and learning**: The Rey Auditory Verbal Learning Test, delayed recall; Spatial Memory: recall the spatial location of dots presented on a screen; **Processing speed:** Choice Reaction Time
Law et al. [48]	Parallel	China	AE *n* = 16 Waitlist control group (CO) *n* = 14	8/8 5/9	77.94 (AE) 75.14 (CO)	Not mentioned	Albert 2011	AE: Type: Structured whole body movement exercise, bicycle, and arm ergometry Intensity: Moderate intensity aerobic exercise, at 4–5/10 on rate of perceived exertion Frequency: 12 sessions Duration: 60-min Time: 8 weeks	**Memory**: Chinese Version Verbal Learning Test; **Processing speed**: Trail Making Test A **Attention**: Trail Making Test B
Combourieu Donnezan et al. [49]	Parallel	France	AE *n* = 18 Cognitive training; Combined training; Control group (CO) *n* = 14	Not mentioned	77.1 (AE) 79.2 (CO)	28.2±0.43; 27.3±0.5	Petersen 2004	AE: Type: Bikes training Intensity: moderate intensity (i.e., 60% HR) Frequency: 2 sessions/week Duration: 60 min Time: 12 weeks	**Executive function**: The Matrix Reasoning test **Attention:** The flexibility part of the Stroop Color Word test, The Digit Span Forward test **Working memory:** The Digit Span Backward test
Damirchi et al. [54]	Parallel	Iran	AE *n* = 11 Mental training; Exercise + mental; Control group (CO) *n* = 9	All female	68.81 (AE) 69.11 (CO)	23.18±2.18; 23.44±2.06	Petersen 2004	Type: Walking with muscular strength training Intensity: Walking from 55% to 75% heart rate reserve, muscle strength rating of perceived exertion within the desired 13 to 15 range; Frequency: 3 sessions/week; Duration: 60 min; Time: 8 weeks	**Processing speed**: Reaction time, Digit Symbol Coding test **Attention**: Modified Stroop color word test error number, Digit span forward subtests of the Wechsler Adult Intelligence Scale

**Table 2:** Characteristics of studies –resistance exercise versus control group

Study	RCT design	Country	Group (N)	Sex (male/female)	Mean age	MMSE	Diagnosis criteria	Exercise	Outcome
Tsai et al. [51]	Parallel	China	Resistance exercise (RE) *n* = 18 Static stretching exercises (CO) *n* = 18	7/11 (RE) 5/13 (CO)	65.44 (RE) 65.17 (CO)	26.56±1.34 (RE) 27.00±1.65 (CO)	Gauthier 2006, Petersen 2004, Winblad 2004	RE: Type: Circuit-exercise use free weights and bodybuilding machines Intensity: 75% of their target 1-RM Frequency: 3 sessions/week Duration: 40 min Time: 16 weeks CO: Type: Static stretching exercises Intensity: No load Frequency, duration, and time: same to RE group	**Working memory**: Digit span of Wechsler-IV Adult intelligence test
Hong et al. [55]	Parallel	Korea	RE *n* = 10 CO *n* = 12	3/7 (RE) 3/9 (CO)	Male 78.33 Female 77.71 (RE) Male 78.33 Female 75.11 (CO)	Not mentioned	Petersen 1999	Type: Elastic band Frequency: 2 sessions/week Intensity: 15-repetition maximum (15RM, about 65% of maximum) Duration: 60 min Time: 12 weeks CO: maintain current lifestyle	**Execution function**: Category/Semantic fluency test, Letter/Phonemic fluency test; **Attention and working memory**: The digit span test; Stroop test; **Immediate Memory and recognition**: Short-term and recognition memory, Rey 15-Item Memory Test
Yoon et al. [57]	Parallel	Korea	RE *n* = 22 CO *n* = 23	6/14 (RE) 7/16 (CO)	73.82 (RE) 74.03 (CO)	24.23±2.89 (RE) 24.22±1.86 (CO)	CDR, Kelaiditi 2013	RE: Type: High-speed resistance training Intensity: blue elastic bands, at a perceived exertion rate of 12–13; 2–3 sets of 12–15 repetitions Frequency: 3 sessions/week Duration: 60 min Time: 16 weeks CO: Type: Balance and resistance band stretching Frequency: 2 sessions/week Duration and time: same with RE group	**Memory**: Rey 15-Item memory test; **Processing speed and attention**: Trail Making A&B Test, **Working memory**: Digit Span (both forward and backward) test; **Executive functions**: Frontal assessment battery
Nagamatsu et al. [53]	Parallel	Canada	Aerobic Training (AE) *n* = 30 RE *n* = 28 Balance and Tone (BAT) *n* = 28	All female	75.6 (AE) 73.9 (RE) 75.1 (CO)	27.4±1.5 (AE) 27.0±1.8 (RE) 27.1±1.7 (CO)	Petersen 2004	Type: Keiser Pressurized Air system Intensity: 6–8 repetitions (two sets), 13–15 on the Borg’s Rating of Perceived Exertion Frequency: 2 sessions/week Duration: 60 min Time: 26 weeks BAT: Type: Stretching exercises, range of motion exercises, balance exercises functional and relaxation techniques Intensity: No load Duration and time: same with RE group	**Verbal memory and learning**: The Rey Auditory Verbal Learning Test, delayed recall; Spatial Memory: recall the spatial location of dots presented on a screen; **Processing speed:** Choice Reaction Time
Lee et al. [56]	Parallel	Korea	RE *n* = 18 Control: lecture consultation (CO) *n* = 22	7/11 9/13	73.7 (RE) 74.2 (CO)	23.8±2.9; 23.4±1.3	CDR Morris 1993	RE: Type: High speed elastic band Frequency:3 sessions/week Intensity:10–12 repetitions of two to three sets, perceived exertion index ranging from 12 to 13 Duration: 50 min Time: 8 weeks	**Executive function**: Frontal Assessment Battery
Lü et al. [58]	Parallel	China	RE *n* = 22 CO *n* = 23	6/16 7/23	69.00 (RE) 70.43 (CO)	7.23±1.63; 26.43±2.00	Petersen, 1999	RE: Type: Momentum based dumbbell-training Intensity: Each individual spinning exercise lasted 1–2 minutes with repetitions set at 4–5 minutes. Frequency: 3 sessions/week Duration: 60 min Time:12-week	**Attention**: The Trail Making Test B; The Digit Span Test forward **Working memory**: The Digit Span Test backward

**Table 3:** Characteristics of studies –Multimodal exercise versus control group

Study	RCT design	Country	Group (*N*)	Sex (male/female)	Mean age	MMSE	Diagnosis criteria	Exercise	Outcome
Lam et al. [59]	Parallel	China	Multimodal motion exercise (ME) *n* = 147; Cognitive group; Integrated cognitive-physical Group; Social group (S) *n* = 131	34/113 (ME) 29/101 (S)	75.5 (ME) 75.4 (S)	25.8±2.3 (ME); 25.6±2.4 (S)	Winblad 2004	AE: Type: One stretching &toning exercise, one mind body exercise and one aerobic exercise session Frequency: 3 sessions/week Duration: 60 min Time: 12 months S: Attending in social activities	**Episodic memory**: The list learning delayed recall test **Executive function**: Category verbal fluency test; Subjective **Cognitive complaints**: Memory Inventory for Chinese

### Risk of bias

Studies without a statement of random sequence generation or allocation concealment in sufficient detail were judged to have an “unclear” risk of bias [45]. Most of the studies did not report protocols; therefore, it was not possible to determine if there was a selection bias. In all the assessed studies, it was not feasible to blind the participants to exercise training interventions. Therefore, the included studies all showed “high risk” of blinding for participants and personnel (performance bias). Half of the included studies stated that non-blind or without details of blinding of outcome assessors. It was judged non-blind as a “high-risk” bias and “not clear” for that without details of the detection bias. Studies were assessed as having a high risk of bias for incomplete outcome data (attrition bias) if they had a relatively high and unbalanced missing rate between groups or did not account for dropout. All other sectors had a low risk of bias (Fig. 2).

#### Aerobic exercise intervention versus control group

Seven studies including 234 participants with MCI relative to aerobic exercise intervention and any active or no intervention control groups were pooled for meta-analyses, which contributed data to no less than one cognitive domain [48–54]. The grouping of cognitive scales and studies over specific domains of cognitive function is shown in Table 4. We were able to perform meta-analyses at all seven of our pre-specified cognitive domains (Supplementary Material, Outcome 1.1 to Outcome 1.7). The results revealed no heterogeneity (I^2^ = 0%) across studies investigating processing speed and recognition, low heterogeneity in immediate memory (I^2^ = 24%), moderate heterogeneity in working memory (I^2^ = 47%), delayed memory (I^2^ = 36%), and attention (I^2^ = 42%). However, studies including executive function demonstrated substantial heterogeneity (I^2^ = 62%). A sensitivity analysis was performed with the main analysis performed with and without each study in investigating executive function; the significance of the results did not change (Supplementary Material, Outcome 1.6 and Outcome 1.6.1). The duration of each aerobic exercise intervention in these studies lasted from 30 to 60 min, with 1–3 sessions/week, ranging from 6 to 26 weeks. There was no significant difference between the aerobic exercise and control groups in immediate memory (*p* = 0.79), executive function (*p* = 0.1), working memory (*p* = 0.49), processing speed (*p* = 0.93), delayed memory (*p* = 0.96), attention (*p* = 0.39), or recognition (*p* = 0.56). One study observed the maintenance effects of aerobic exercise after treatment termination, reporting that the treatment effects declined during the treatment-free period [52]. There was no difference between the aerobic exercise and control groups in dropout rates (odds ratio [OR], 2.47; 95% CI, 0.94–6.49; seven trials, 251 participants; Supplementary Material, Outcome 1.8). Two studies reported no adverse events in any group during the course of the intervention [48, 50]. One study reported the presence of adverse events, including episodes of shortness of breath that resolved with rest and non-injurious falls [53], with no significant differences between groups (*p* = 0.54). The other studies did not mention any adverse events.

**Table 4:** Grouping of cognitive tests and studies over cognitive functions

Cognitive function	Cognitive tests	Trial
Immediate memory	The Verbal Learning and Memory Test Direct recall	Scherder et al. [52]
	Chinese Version Verbal Learning Test, total free recall	Law et al. [48]
	The Rey Auditory Verbal Learning Test	Nagamatsu et al. [53], Yogev-Seligmann et al. [50]
	Rey 15-Item Memory Test	Hong et al. [55], Yoon et al. [57]
Working memory	Digit span backward	Lu et al. [58], Combourieu Donnezan et al. [49], Hong et al. [55]
	Digit span component of the Wechsler memory test	Scherder et al. [52], Yoon et al. [57], Tsai et al. [51], Yogev-Seligmann et al. [50]
Delayed memory	Rey auditory verbal learning test delayed recall trail	Nagamatsu et al. [53], Yogev-Seligmann et al. [50]
	The Verbal Learning and Memory Test, delayed recall	Scherder et al. [52]
	Chinese Version Verbal Learning Test, delayed recall	Law et al. [48]
	The list learning delayed recall test	Lam et al. [59]
Processing speed	Reaction time of modified Stroop color-word test	Damirchi et al. [54]
	Reaction time of spatial memory	Nagamatsu et al. [53]
	Trail making test A	Yoon et al. [57], Law et al. [48], Yogev-Seligmann et al. [50]
	Trail-making A + B	Scherder et al. [52]
Attention	Trail making test B	Lu et al. [58], Yoon et al. [57], Law et al. [48], Yogev-Seligmann et al. [50]
	Digit span (forward)	Combourieu Donnezan et al. [49], Damirchi et al. [54], Hong et al. [55]
Executive function	Category verbal fluency	Scherder et al. [52], Lam et al. [59]
	Verbal fluency, semantic	Hong et al. [55], Yogev-Seligmann et al. [50]
	Frontal Assessment Battery	Yoon et al. [57], Lee et al. [56]
	Matrix Reasoning test	Combourieu Donnezan et al. [49]
Recognition	Faces/houses recognition	Yogev-Seligmann et al. [50]
	The Verbal Learning and Memory Test, recognition	Scherder et al. [52]
	Rey 15-Item Memory Test, recognition	Hong et al. [55]

#### Resistance exercise intervention versus control group

**Fig. 3:**
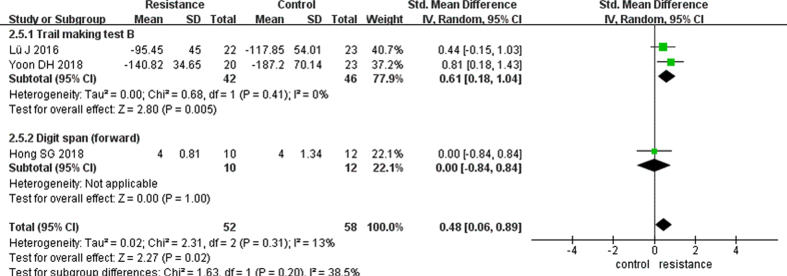
Outcome 2.5 Impact of resistance exercise training on attention. CI, confidence interval; IV, inverse variance; SD standard deviation; Std, standardized.

**Fig. 4:**
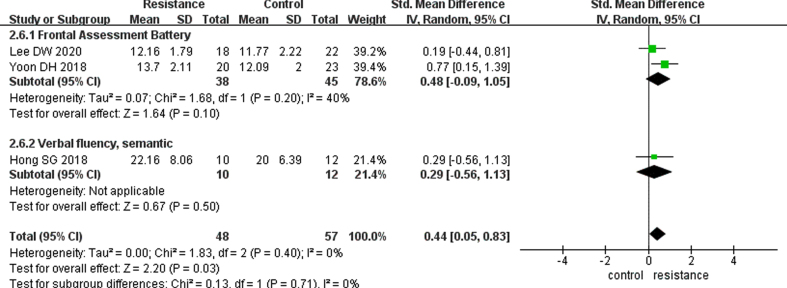
Outcome 2.6 Impact of resistance exercise training on executive function. CI, confidence interval; IV, inverse variance; SD standard deviation; Std, standardized.

Six studies including 242 participants with MCI relative to resistance exercise intervention and any active or no intervention control groups were pooled for meta-analyses, which contributed data to at least one cognitive domain [51, 53, 55–58]. We performed meta-analyses on five cognitive domains because only one study was involved in the domain of delayed memory [53] and recognition [55] each (Figs. 3 and 4; Supplementary Material, Outcome 2.1 to Outcome 2.4, Outcome 2.7). The results revealed no heterogeneity (I^2^ = 0%) across studies investigating immediate memory and executive function and low heterogeneity in attention (I^2^ = 13%); however, studies including working memory (I^2^ = 32%) and processing speed (I^2^ = 42%) demonstrated moderate heterogeneity.

The duration of each resistance exercise intervention in these studies lasted from 40 to 60 minutes, with 2–3 sessions/week, ranging from 8 to 26 weeks. Meta-analysis on pooled data revealed significant improvement differences for the resistance exercise intervention versus control groups on measures of attention (0.48 [0.06–0.89], *p* < 0.05) and executive function (0.44 [0.05–0.83], *p* < 0.05), but not on immediate memory (*p* = 0.79), working memory (*p* = 0.19), processing speed (*p* = 0.92), delayed memory (*p* = 0.82), or recognition (*p* = 0.77). There was no difference in the dropout rates between the resistance exercise and control groups (OR, 1.21; 95% CI, 0.59–2.50; six trials; Supplementary Material, Outcome 2.8). One study reported the maintenance effects of resistance exercise treatment on participants with MCI [49], and the outcome of the matrix reasoning test (*p* < 0.01) and digit span forward (*p* < 0.05) still improved for the 6-month post-test relative to the pre-test. Two studies reported no adverse events in any group during the course of the intervention [56, 58]. One study reported no significant differences of adverse event occurrence between groups (*p* = 0.54) [53]. The other studies did not mention any adverse events.

#### Multimodal exercise intervention versus control group

Lam et al. assessed the effect of multimodal exercise intervention on cognitive function in 114 individuals with MCI [59]. These cognitive domains involved outcomes of pre- and post-training measures of delayed recall and executive function (category verbal fluency test). The trial had a fair methodological quality. The author found a significant improvement favoring multimodal exercise intervention over the control group on measures of both the category verbal fluency test and delayed recall after 12 months of intervention (multilevel model, *p* < 0.001). There was no difference between the multimodal exercise and control groups in the dropout rates. This study reported no adverse events associated with this intervention. No differences in age and educational level were observed between completers and defaulters.

## DISCUSSION

### Summary of main results

We examined the effects of aerobic, resistance, and multimodal exercise training on cognitive function in adults aged > 60 years with MCI. One multimodal exercise intervention study was presented insufficiently. Meta-analysis results revealed that, compared to the control group, resistance exercise significantly improved performance on measures of attention and executive function. No significant differences were observed in any of the remaining cognitive domains.

We included 12 studies after screening because of a more qualified inclusion criterion. Most studies involved small sample sizes. There was significant heterogeneity in the methodologies across the studies involved, including sample size, exercise schemes, participant compliance, and study quality. Although the review analyzed the fine-sorted subgroups of cognitive function, there could be variations in the results of each measure. We observed that half of the studies reported significant improvements in composite measures of cognitive function for exercise versus controls, including executive function [49, 52, 57], working memory [55], and processing speed [50, 52, 53, 57]. Despite this, the majority of comparisons yielded no significant differences. Additionally, the results of this meta-analysis could have been affected by the quality of the included trials. Across the included papers, approximately half did not state the methods of randomization or blinding of outcome assessors. For all but one trial, allocation was poorly reported. No trial evaluated contamination bias that could have interfered with the outcomes. Only two trials reported protocols [57, 59]; for most trials, it was not feasible to determine whether there was selective reporting of results.

Some researchers have suggested that improvements in cognitive function could be attributed to improvements in cardiovascular fitness due to exercise [63]. Changes in grip strength and walking speed have been correlated with mental state and fluid cognition [64]. However, most trials involved in this review did not report any objective measures of cardiorespiratory fitness. In addition, studies have reported that short session duration and higher frequency might predict higher effect sizes [65, 66]. However, owing to the inconsistency of exercise modalities in this review, dose-response tests were not performed in this study.

### Agreements and disagreements with other studies or reviews

Seven meta-analyses published data based on similar hypotheses yet failed to find comparable results.

Gates et al. assessed the effects of exercise on cognitive function in 14 RCT trials with 1,695 participants [43]. Their eligibility criteria differed from this study in that they included participants with probable MCI, and subjective memory decline who were aged > 65. We both set the criteria for exercise intervention up longer than 4 weeks and prescribed them specifically. However, we only included trials that reported exercise training effectively supervised by centers, physicians, or professionals; those self-reported, at home, or without reported supervision were excluded. The authors concluded that there is limited evidence to prove that any exercise modality improves cognitive function in individuals with MCI.

De Souto Barreto et al. reviewed five papers on long-term exercise (12 months or longer) in older adults with MCI, onset of dementia, or clinically meaningful cognitive decline [67]. Apart from the inclusion criteria, another difference is that they considered the change in MMSE score as a main outcome indicator and analyzed the overall effect of exercise, whereas we discussed the different exercise modalities separately. Their review observed no significant effects of exercise, either individually or in combination, on reducing the risk of cognitive decline.

Zhang et al. published a meta-analytic review of traditional Chinese exercise in older adults with MCI [68]. They found that four Tai Chi studies and one Liuzijue study met the inclusion criteria. We excluded trials on participants with MCI with a definite etiology, whereas this review included medical or neurological disorders, such as AD, dementia, or Parkinson’s disease. The authors concluded that exercise improved the visuospatial function for individuals with MCI.

The meta-analysis presented by Lee et al. has a similar aim (reporting the effects of exercise intervention for older adults with MCI) and exclusion criteria (cross-sectional, protocol, and review studies) [69]. In contrast to this review, our study subdivided cognitive function into detailed items instead of MMSE. In addition, this review finally involved three eligible studies, including five exercise interventions (aerobic or resistance exercise) combined for data analysis, and only one study was classified into a functional category. This review reported that cognitive function was significantly increased in the exercise group.

Biazus-Sehn et al. reviewed the global effects of various exercise modalities on MCI. Their search identified 27 studies that reported data [42]. The difference is that they involved participants with a mean age of ≥60 years who had other diseases, such as insulin resistance. We excluded those who were not purely cognitively impaired but had other diseases, which might disturb the exercise effect and cognitive function. They reported improved global cognitive function, executive function, and delayed recall after the exercise.

Similarly, Zhu et al. reviewed and analyzed aerobic dance in participants with MCI aged > 50 years. This meta-analysis involved five studies with 842 patients [70]. They found that aerobic dance, including Tai Chi, improved global cognitive function and memory executive function in older adults with MCI.

A meta-analysis by Lin et al. assessed the effects of Tai Chi on cognitive performance. Except for exercise intervention, the criteria differed from this review in including younger participants with MCI [71]. This meta-analysis indicated that Tai Chi has positive clinical effects on cognitive function (global cognitive function, memory and learning, executive function) in older adults with MCI.

### Implications for practice and research

We found that resistance exercise improved executive function and attention performance in patients aged > 60 years. However, there might be some confounding factors in the outcomes. First, there are more studies and diversified scales involved in aerobic exercise analyses, which might lead to higher heterogeneity, and the large variability in training paradigms and study quality might also influence the outcome. Further investigations into the parameters and populations most associated with the efficacy of physical training for cognitive function are necessary. Cognition is a complex and multi-class function; however, there is insufficient evidence to illustrate the relationship between exercise and brain-processed cognitive function. Certainly, clear differences exist in the effects of exercise on the cognition subgroups. Within the seven disparate intrinsic connectivity networks in the brain, the beneficial effects exerted by aerobic exercise might be mediated by the greatest association with the executive and dorsal attention networks [72]. Exercise activates the hub region of the executive network (e.g., attention, working memory, cognitive control) in the brain [11, 73], and the duration of exercise may be positively correlated with its volume [74]. Exercise-induced brain functional homogeneity variation is likely to improve executive control behavior and predict attention behavior [75]. This evidence may help to explain the results. Thus, to elucidate the exact relationship between exercise and cognitive function in patients with MCI, more large-sample RCTs and animal studies are required in the future. It also raises our wider understanding of how to improve exercise compliance and to use a smaller battery of cognitive scales for the uniformity and comparability of results.

## Supplementary Material

Supplementary Material
