# Supplementary material for: Exercise Training for Mild Cognitive Impairment Adults Older Than 60: A Systematic Review and Meta-Analysis
Source: J Alzheimers Dis. 2022 Aug 16;88(4):1263–78. doi: 10.3233/JAD-220243 (PMC9484098; doi:10.3233/JAD-220243)
Supplement: Supplementary Material [file jad-88-jad220243-s001.pdf]

Supplementary Material

Exercise Training for Mild Cognitive Impairment Adults Older Than 60: A Systematic Review and Meta-Analysis

Section and Topic	Item #	Checklist item	Location where item is reported
**TITLE**			
Title	1	Identify the report as a systematic review.	1
**ABSTRACT**			
Abstract	2	See the PRISMA 2020 for Abstracts checklist.	2
**INTRODUCTION**			
Rationale	3	Describe the rationale for the review in the context of existing knowledge.	3-4
Objectives	4	Provide an explicit statement of the objective(s) or question(s) the review addresses.	4
**METHODS**			
Eligibility criteria	5	Specify the inclusion and exclusion criteria for the review and how studies were grouped for the syntheses.	5-6
Information sources	6	Specify all databases, registers, websites, organisations, reference lists and other sources searched or consulted to identify studies. Specify the date when each source was last searched or consulted.	4-5
Search strategy	7	Present the full search strategies for all databases, registers and websites, including any filters and limits used.	5, Supplementary Material
Selection process	8	Specify the methods used to decide whether a study met the inclusion criteria of the review, including how many reviewers screened each record and each report retrieved, whether they worked independently, and if applicable, details of automation tools used in the process.	6
Data collection process	9	Specify the methods used to collect data from reports, including how many reviewers collected data from each report, whether they worked independently, any processes for obtaining or confirming data from study investigators, and if applicable, details of automation tools used in the process.	6
Data items	10	List and define all outcomes for which data were sought. Specify whether all results that were compatible with each outcome domain in each study were sought (e.g. for all measures, time points, analyses), and if not, the methods used to decide which results to collect.	6
Study risk of bias assessment	11	Specify the methods used to assess risk of bias in the included studies, including details of the tool(s) used, how many reviewers assessed each study and whether they worked independently, and if applicable, details of automation tools used in the process.	7
Effect measures	12	Specify for each outcome the effect measure(s) (e.g. risk ratio, mean difference) used in the synthesis or presentation of results.	8
Synthesis methods	13	Describe the processes used to decide which studies were eligible for each synthesis (e.g. tabulating the study intervention characteristics and comparing against the planned groups for each synthesis (item #5)).	6-7
Reporting bias assessment	14	Describe any methods used to assess risk of bias due to missing results in a synthesis (arising from reporting biases).	7
Certainty assessment	15	Describe any methods used to assess certainty (or confidence) in the body of evidence for an outcome.	7

Section and Topic	Item #	Checklist item	Location where item is reported
**RESULTS**			
Study selection	16a	Describe the results of the search and selection process, from the number of records identified in the search to the number of studies included in the review, ideally using a flow diagram.	31
	16b	Cite studies that might appear to meet the inclusion criteria, but which were excluded, and explain why they were excluded.	8
Study characteristics	17	Cite each included study and present its characteristics.	24-28
Risk of bias in studies	18	Present assessments of risk of bias for each included study.	32
Results of individual studies	19	For all outcomes, present, for each study: (a) summary statistics for each group (where appropriate) and (b) an effect estimate and its precision (e.g. confidence/credible interval), ideally using structured tables or plots.	33, Supplementary Material
Results of syntheses	20a	For each synthesis, briefly summarise the characteristics and risk of bias among contributing studies.	9
	20b	Present results of all statistical syntheses conducted. If meta-analysis was done, present for each the summary estimate and its precision (e.g., confidence/credible interval) and measures of statistical heterogeneity. If comparing groups, describe the direction of the effect.	33, Supplementary Material
Reporting biases	21	Present assessments of risk of bias due to missing results (arising from reporting biases) for each synthesis assessed.	9
Certainty of evidence	22	Present assessments of certainty (or confidence) in the body of evidence for each outcome assessed.	9-11
**DISCUSSION**			
Discussion	23a	Provide a general interpretation of the results in the context of other evidence.	12-13
	23b	Discuss any limitations of the evidence included in the review.	13
	23c	Discuss any limitations of the review processes used.	13
	23d	Discuss implications of the results for practice, policy, and future research.	15
**OTHER INFORMATION**			
Registration and protocol	24a	Provide registration information for the review, including register name and registration number, or state that the review was not registered.	4
Support	25	Describe sources of financial or non-financial support for the review, and the role of the funders or sponsors in the review.	17
Competing interests	26	Declare any competing interests of review authors.	17

Search strategy on PubMed (www.ncbi.nlm.nih.gov) on November 2, 2021.
(1800 - 2021)

(((((((((((((("**Exercise**"[Mesh]) OR (((((((((((((((((((((((((Exercises[Title/Abstract]) OR (Physical Activity[Title/Abstract])) OR (Activities, Physical[Title/Abstract])) OR (Activity, Physical[Title/Abstract])) OR (Physical Activities[Title/Abstract])) OR (Exercise, Physical[Title/Abstract])) OR (Exercises, Physical[Title/Abstract])) OR (Physical Exercise[Title/Abstract])) OR (Physical Exercises[Title/Abstract])) OR (Acute Exercise[Title/Abstract])) OR (Acute Exercises[Title/Abstract])) OR (Exercise, Acute[Title/Abstract])) OR (Exercises, Acute[Title/Abstract])) OR (Exercise, Isometric[Title/Abstract])) OR (Exercises, Isometric[Title/Abstract])) OR (Isometric Exercises[Title/Abstract])) OR (Isometric Exercise[Title/Abstract])) OR (Exercise, Aerobic[Title/Abstract])) OR (Aerobic Exercise[Title/Abstract])) OR (Aerobic Exercises[Title/Abstract])) OR (Exercises, Aerobic[Title/Abstract])) OR (Exercise Training[Title/Abstract])) OR (Exercise Trainings[Title/Abstract])) OR (Training, Exercise[Title/Abstract])) OR (Trainings, Exercise[Title/Abstract])))) OR (("**Exercise Therapy**"[Mesh]) OR (((((((((((Remedial Exercise[Title/Abstract]) OR (Exercise, Remedial[Title/Abstract])) OR (Exercises, Remedial[Title/Abstract])) OR (Remedial Exercises[Title/Abstract])) OR (Therapy, Exercise[Title/Abstract])) OR (Exercise Therapies[Title/Abstract])) OR (Therapies, Exercise[Title/Abstract])) OR (Rehabilitation Exercise[Title/Abstract])) OR (Exercise, Rehabilitation[Title/Abstract])) OR (Exercises, Rehabilitation[Title/Abstract])) OR (Rehabilitation Exercises[Title/Abstract])))) OR (("**Resistance Training**"[Mesh]) OR (((Training, Resistance[Title/Abstract]) OR (Strength Training[Title/Abstract])) OR (Training, Strength[Title/Abstract])))) OR (("**Tai Ji**"[Mesh]) OR ((((((((((Tai-ji[Title/Abstract]) OR (Tai Chi[Title/Abstract])) OR (Chi, Tai[Title/Abstract])) OR (Tai Ji Quan[Title/Abstract])) OR (Ji Quan, Tai[Title/Abstract])) OR (Quan, Tai Ji[Title/Abstract])) OR (Taiji[Title/Abstract])) OR (Taijiquan[Title/Abstract])) OR (T'ai Chi[Title/Abstract])) OR (Tai Chi Chuan[Title/Abstract])))) OR (("**Yoga**"[Mesh]) OR ("**walking**"[Mesh]) OR ("**jogging**"[Mesh]) OR ("**running**"[Mesh]) OR ("**swimming**"[Mesh]) OR ("**Bicycling**"[Mesh]))) OR (("**Sports**"[Mesh]) OR (((sport[Title/Abstract]) OR (Athletics[Title/Abstract])) OR (Athletic[Title/Abstract])))) OR (("**Circuit-Based Exercise**"[Mesh]) OR ((((((Circuit Based Exercise[Title/Abstract]) OR (Circuit-Based Exercises[Title/Abstract])) OR (Exercise, Circuit-Based[Title/Abstract])) OR (Exercises, Circuit-Based[Title/Abstract])) OR (Circuit Training[Title/Abstract])) OR (Training, Circuit[Title/Abstract])))) OR (("**Physical Fitness**"[Mesh]) OR ((Fitness, Physical[Title/Abstract]) OR (Fitness[Title/Abstract]) OR (cardiorespiratory fitness [Title/Abstract]) OR (Fitness, Cardiorespiratory [Title/Abstract])))) OR (("**Endurance Training**"[Mesh]) OR (Training, Endurance[Title/Abstract]))) OR (("**Muscle Stretching Exercises**"[Mesh]) OR (((((((((((((((((((((((((((((((((Exercise, Muscle Stretching[Title/Abstract]) OR (Muscle Stretching Exercise[Title/Abstract])) OR (Static Stretching[Title/Abstract])) OR (Stretching, Static[Title/Abstract])) OR (Active Stretching[Title/Abstract])) OR (Stretching, Active[Title/Abstract])) OR (Static-Active Stretching[Title/Abstract])) OR (Static Active Stretching[Title/Abstract])) OR (Stretching, Static-Active[Title/Abstract])) OR (Isometric Stretching[Title/Abstract])) OR (Stretching, Isometric[Title/Abstract])) OR (Ballistic Stretching[Title/Abstract])) OR (Stretching, Ballistic[Title/Abstract])) OR (Dynamic Stretching[Title/Abstract])) OR (Stretching, Dynamic[Title/Abstract])) OR (Proprioceptive Neuromuscular Facilitation (PNF) Stretching[Title/Abstract])) OR (PNF Stretching[Title/Abstract])) OR (PNF Stretchings[Title/Abstract])) OR (Stretching, PNF[Title/Abstract])) OR (PNF Stretching Exercise[Title/Abstract])) OR (Exercise, PNF Stretching[Title/Abstract])) OR (PNF Stretching Exercises[Title/Abstract])) OR (Stretching Exercise, PNF[Title/Abstract])) OR (Proprioceptive Neuromuscular Facilitation[Title/Abstract])) OR (Neuromuscular Facilitation, Proprioceptive[Title/Abstract])) OR (Proprioceptive Neuromuscular Facilitations[Title/Abstract])) OR (Passive Stretching[Title/Abstract])) OR (Stretching, Passive[Title/Abstract])) OR (Relaxed Stretching[Title/Abstract])) OR (Stretching, Relaxed[Title/Abstract])) OR (Static-Passive Stretching[Title/Abstract])) OR (Static Passive Stretching[Title/Abstract])) OR (Stretching, Static-Passive[Title/Abstract])))) OR (("**Plyometric Exercise**"[Mesh]) OR ((((((((((((((((((((((((((((Exercise, Plyometric[Title/Abstract]) OR (Exercises, Plyometric[Title/Abstract])) OR (Plyometric Exercises[Title/Abstract])) OR (Plyometric Drill[Title/Abstract])) OR (Drill, Plyometric[Title/Abstract])) OR (Drills, Plyometric[Title/Abstract])) OR (Plyometric Drills[Title/Abstract])) OR (Plyometric Training[Title/Abstract])) OR (Plyometric Trainings[Title/Abstract])) OR (Training, Plyometric[Title/Abstract])) OR (Trainings, Plyometric[Title/Abstract])) OR (Stretch-

Shortening Exercise[Title/Abstract])) OR (Exercise, Stretch-Shortening[Title/Abstract])) OR (Exercises, Stretch-Shortening[Title/Abstract])) OR (Stretch Shortening Exercise[Title/Abstract])) OR (Stretch-Shortening Exercises[Title/Abstract])) OR (Stretch-Shortening Cycle Exercise[Title/Abstract])) OR (Cycle Exercise, Stretch-Shortening[Title/Abstract])) OR (Cycle Exercises, Stretch-Shortening[Title/Abstract])) OR (Exercise, Stretch-Shortening Cycle[Title/Abstract])) OR (Exercises, Stretch-Shortening Cycle[Title/Abstract])) OR (Stretch Shortening Cycle Exercise[Title/Abstract])) OR (Stretch-Shortening Cycle Exercises[Title/Abstract])) OR (Stretch-Shortening Drill[Title/Abstract])) OR (Drill, Stretch-Shortening[Title/Abstract])) OR (Drills, Stretch-Shortening[Title/Abstract])) OR (Stretch Shortening Drill[Title/Abstract])) OR (Stretch-Shortening Drills[Title/Abstract]))))) AND (("**Cognitive Dysfunction**"[Mesh]) OR (((((((((((((((((((((((((((Cognitive Dysfunctions[Title/Abstract]) OR (Dysfunction, Cognitive[Title/Abstract])) OR (Dysfunctions, Cognitive[Title/Abstract])) OR (Cognitive Impairments[Title/Abstract])) OR (Cognitive Impairment[Title/Abstract])) OR (Impairment, Cognitive[Title/Abstract])) OR (Impairments, Cognitive[Title/Abstract])) OR (Mild Cognitive Impairment[Title/Abstract])) OR (Cognitive Impairment, Mild[Title/Abstract])) OR (Cognitive Impairments, Mild[Title/Abstract])) OR (Impairment, Mild Cognitive[Title/Abstract])) OR (Impairments, Mild Cognitive[Title/Abstract])) OR (Mild Cognitive Impairments[Title/Abstract])) OR (Mild Neurocognitive Disorder[Title/Abstract])) OR (Disorder, Mild Neurocognitive[Title/Abstract])) OR (Disorders, Mild Neurocognitive[Title/Abstract])) OR (Mild Neurocognitive Disorders[Title/Abstract])) OR (Neurocognitive Disorder, Mild[Title/Abstract])) OR (Neurocognitive Disorders, Mild[Title/Abstract])) OR (Cognitive Decline[Title/Abstract])) OR (Cognitive Declines[Title/Abstract])) OR (Decline, Cognitive[Title/Abstract])) OR (Declines, Cognitive[Title/Abstract])) OR (Mental Deterioration[Title/Abstract])) OR (Deterioration, Mental[Title/Abstract])) OR (Deteriorations, Mental[Title/Abstract])) OR (Mental Deteriorations[Title/Abstract]))))) AND ((("**Aged**"[Mesh]) OR ((((((older adults[Title/Abstract]) OR (elder[Title/Abstract])) OR (senior[Title/Abstract])) OR (older[Title/Abstract])) OR (aging[Title/Abstract])) OR (elderly[Title/Abstract])))) OR (((((((((("**Aged, 80 and over**"[Mesh]) OR (Nonagenarians[Title/Abstract])) OR (Nonagenarian[Title/Abstract])) OR (Oldest Old[Title/Abstract])) OR (Centenarians[Title/Abstract])) OR (Centenarian[Title/Abstract])) OR (Octogenarian[Title/Abstract])) OR (Octogenarians[Title/Abstract])) OR (very elderly[Title/Abstract])) OR (very old[Title/Abstract])))) AND (randomized controlled trial[Publication Type] OR randomized[Title/Abstract] OR placebo[Title/Abstract])

Search strategy on Embase on November 3, 2021

Embase <1974 to 2021 November 3>

#	Query	Results from 3 November 2021
1	'exercise'/exp	388,292
2	'exercises':ab,ti OR 'physical activity':ab,ti OR 'activities, physical':ab,ti OR 'activity, physical':ab,ti OR 'physical activities':ab,ti OR 'exercise, physical':ab,ti OR 'exercises, physical':ab,ti OR 'physical exercise':ab,ti OR 'physical exercises':ab,ti OR 'acute exercise':ab,ti OR 'acute exercises':ab,ti OR 'exercise, acute':ab,ti OR 'exercises, acute':ab,ti OR 'exercises, isometric':ab,ti OR 'exercise, isometric':ab,ti OR 'isometric exercises':ab,ti OR 'isometric exercise':ab,ti OR 'exercise, aerobic':ab,ti OR 'aerobic exercise':ab,ti OR 'aerobic exercises':ab,ti OR 'exercises, aerobic':ab,ti OR 'exercise trainings':ab,ti OR 'training, exercise':ab,ti OR 'trainings, exercise':ab,ti OR 'exercise training':ab,ti OR 'exertion':ab,ti OR 'fitness training':ab,ti OR 'fitness workout':ab,ti OR 'physical effort':ab,ti OR 'physical exertion':ab,ti OR 'physical work-out':ab,ti OR 'physical workout':ab,ti OR 'training, physical':ab,ti	302,600
3	#1 OR #2	557,826
4	'circuit training'/exp	293
5	'circuit based exercise':ab,ti OR 'circuit-based training':ab,ti OR 'circuit-based exercises':ab,ti OR 'exercise, circuit-based':ab,ti OR 'exercises, circuit-based':ab,ti OR 'circuit-based exercise':ab,ti OR 'training, circuit':ab,ti OR 'circuit-type training':ab,ti OR 'circuit-type exercise':ab,ti	52
6	#4 OR #5	333
7	'kinesiotherapy'/exp	90,605
8	'exercise therapy':ab,ti OR 'remedial exercise':ab,ti OR 'exercise, remedial':ab,ti OR 'exercises, remedial':ab,ti OR 'remedial exercises':ab,ti OR 'therapy, exercise':ab,ti OR 'exercise therapies':ab,ti OR 'therapies, exercise':ab,ti OR 'rehabilitation exercise':ab,ti OR 'exercise, rehabilitation':ab,ti OR 'exercises, rehabilitation':ab,ti OR 'rehabilitation exercises':ab,ti OR 'treatment, exercise':ab,ti OR 'therapeutic exercise':ab,ti OR 'exercise treatment':ab,ti	11,400
9	#7 OR #8	95,410
10	'resistance training'/exp	22,072
11	'resistance exercise':ab,ti OR 'resistance exercise training':ab,ti OR 'resistance-type exercise':ab,ti OR 'resistance-type training':ab,ti OR 'strength-type exercise':ab,ti OR 'strength training':ab,ti OR 'strength-type training':ab,ti OR 'training, resistance':ab,ti OR 'training, strength':ab,ti OR 'strength exercise':ab,ti	14,504
12	#10 OR #11	27,830
13	'tai chi'/exp	3,382
14	'tai chi chuan':ab,ti OR 'chi, tai':ab,ti OR 'tai ji quan':ab,ti OR 'ji quan, tai':ab,ti OR 'quan, tai ji':ab,ti OR 'taiji':ab,ti OR 'taijiquan':ab,ti OR 'tai ji':ab,ti OR 'tai chi':ab,ti	2,899
15	#13 OR #14	3,888
16	'sport'/exp OR 'sports':ab,ti	230,258
17	'fitness'/exp	40,988
18	'fitness, physical':ab,ti OR 'physical fitness':ab,ti OR 'cardiorespiratory fitness':ab,ti OR 'fitness, cardiorespiratory':ab,ti	19,993
19	#17 OR #18	49,244
20	'endurance training'/exp	7,651
21	'endurance exercise':ab,ti OR 'endurance exercise training':ab,ti OR 'training, endurance':ab,ti	5,648

22	#20 OR #21	11,077
23	'jogging'/exp OR 'walking'/exp OR 'swimming'/exp OR 'running'/exp OR 'bicycling'/exp OR 'yoga'/exp	205,811
24	'stretching exercise'/exp	3,859
25	'muscle stretching exercises':ab,ti OR 'stretching exercises':ab,ti OR 'exercise, muscle stretching':ab,ti OR 'muscle stretching exercise':ab,ti OR 'static stretching':ab,ti OR 'stretching, static':ab,ti OR 'active stretching':ab,ti OR 'stretching, active':ab,ti OR 'static-active stretching':ab,ti OR 'static active stretching':ab,ti OR 'stretching, static-active':ab,ti OR 'isometric stretching':ab,ti OR 'stretching, isometric':ab,ti OR 'ballistic stretching':ab,ti OR 'stretching, ballistic':ab,ti OR 'dynamic stretching':ab,ti OR 'stretching, dynamic':ab,ti OR 'proprioceptive neuromuscular facilitation (pnf) stretching':ab,ti OR 'pnf stretching':ab,ti OR 'pnf stretchings':ab,ti OR 'stretching, pnf':ab,ti OR 'pnf stretching exercise':ab,ti OR 'exercise, pnf stretching':ab,ti OR 'pnf stretching exercises':ab,ti OR 'stretching exercise, pnf':ab,ti OR 'proprioceptive neuromuscular facilitation':ab,ti OR 'neuromuscular facilitation, proprioceptive':ab,ti OR 'proprioceptive neuromuscular facilitations':ab,ti OR 'passive stretching':ab,ti OR 'stretching, passive':ab,ti OR 'relaxed stretching':ab,ti OR 'stretching, relaxed':ab,ti OR 'static-passive stretching':ab,ti OR 'static passive stretching':ab,ti OR 'stretching, static-passive':ab,ti	3,190
26	#24 OR #25	5,771
27	'aged'/exp	3,387,213
28	'senium':ab,ti OR 'older adults':ab,ti OR 'elder':ab,ti OR 'senior':ab,ti OR 'older':ab,ti OR 'aging':ab,ti OR 'elderly':ab,ti	1,200,290
29	#27 OR #28	4,018,271
30	'very elderly'/exp	242,898
31	'aged, 80 and over':ab,ti OR 'very old':ab,ti OR 'octogenarians':ab,ti OR 'nonagenarians':ab,ti OR 'nonagenarian':ab,ti OR 'centenarians':ab,ti OR 'centenarian':ab,ti OR 'octogenarian':ab,ti OR 'oldest old':ab,ti	18,530
32	#30 OR #31	253,770
33	'mild cognitive impairment'/exp	30,795
34	'cognitive dysfunction':ab,ti OR 'cognitive dysfunctions':ab,ti OR 'dysfunction, cognitive':ab,ti OR 'dysfunctions, cognitive':ab,ti OR 'cognitive impairments':ab,ti OR 'cognitive impairment':ab,ti OR 'impairment, cognitive':ab,ti OR 'impairments, cognitive':ab,ti OR 'cognitive impairment, mild':ab,ti OR 'cognitive impairments, mild':ab,ti OR 'impairment, mild cognitive':ab,ti OR 'impairments, mild cognitive':ab,ti OR 'mild cognitive impairments':ab,ti OR 'mild neurocognitive disorder':ab,ti OR 'disorder, mild neurocognitive':ab,ti OR 'disorders, mild neurocognitive':ab,ti OR 'mild neurocognitive disorders':ab,ti OR 'neurocognitive disorder, mild':ab,ti OR 'neurocognitive disorders, mild':ab,ti OR 'cognitive decline':ab,ti OR 'cognitive declines':ab,ti OR 'decline, cognitive':ab,ti OR 'declines, cognitive':ab,ti OR 'mental deterioration':ab,ti OR 'deterioration, mental':ab,ti OR 'deteriorations, mental':ab,ti OR 'mental deteriorations':ab,ti	161,303
35	#33 OR #34	166,239
36	'randomized controlled trial':ab,ti OR 'randomized':ab,ti OR 'placebo':ab,ti	1,005,320
37	#3 OR #6 OR #9 OR #12 OR #15 OR #16 OR #19 OR #22 OR #23 OR #26	868,531
38	#29 OR #32	4,020,391
39	#35 AND #36 AND #37 AND #38	865

Search strategy on Cochrane on November 5, 2021

#	Search
1	MeSH descriptor: [Exercise] explode all trees
2	(Exercises):ti,ab,kw OR (Physical Activity):ti,ab,kw OR (Activities, Physical):ti,ab,kw OR (Activity, Physical):ti,ab,kw OR (Physical Activities):ti,ab,kw OR (Exercise, Physical):ti,ab,kw OR (Exercises, Physical):ti,ab,kw OR (Physical Exercise):ti,ab,kw OR (Physical Exercises):ti,ab,kw OR (Acute Exercise):ti,ab,kw OR (Acute Exercises):ti,ab,kw OR (Exercise, Acute):ti,ab,kw OR (Exercises, Acute):ti,ab,kw OR (Exercise, Isometric):ti,ab,kw OR (Exercises, Isometric):ti,ab,kw OR (Isometric Exercises):ti,ab,kw OR (Isometric Exercise):ti,ab,kw OR (Exercise, Aerobic):ti,ab,kw OR (Aerobic Exercise):ti,ab,kw OR (Aerobic Exercises):ti,ab,kw OR (Exercises, Aerobic):ti,ab,kw OR (Exercise Training):ti,ab,kw OR (Exercise Trainings):ti,ab,kw OR (Training, Exercise):ti,ab,kw OR (Trainings, Exercise):ti,ab,kw OR (exercise training):ti,ab,kw OR (exertion):ti,ab,kw OR (fitness training):ti,ab,kw OR (fitness workout):ti,ab,kw OR (physical effort):ti,ab,kw OR (physical exertion):ti,ab,kw OR (physical work-out):ti,ab,kw OR (physical workout):ti,ab,kw OR (training, physical):ti,ab,kw
3	#1 OR #2
4	MeSH descriptor: [Exercise Therapy] explode all trees
5	(kinesiotherapy):ti,ab,kw OR (Remedial Exercise):ti,ab,kw OR (Exercise, Remedial):ti,ab,kw OR (Exercises, Remedial):ti,ab,kw OR (Remedial Exercises):ti,ab,kw OR (Therapy, Exercise):ti,ab,kw OR (Exercise Therapies):ti,ab,kw OR (Therapies, Exercise):ti,ab,kw OR (Rehabilitation Exercise):ti,ab,kw OR (Exercise, Rehabilitation):ti,ab,kw OR (Exercises, Rehabilitation):ti,ab,kw OR (Rehabilitation Exercises):ti,ab,kw OR (treatment, exercise):ti,ab,kw OR (exercise treatment):ti,ab,kw OR (therapeutic exercise):ti,ab,kw
6	#4 OR #5
7	MeSH descriptor: [Circuit-Based Exercise] explode all trees
8	(Circuit Based Exercise):ti,ab,kw OR (circuit-based training):ti,ab,kw OR (Circuit-Based Exercises):ti,ab,kw OR (Exercise, Circuit-Based):ti,ab,kw OR (Exercises, Circuit-Based):ti,ab,kw OR (circuit training):ti,ab,kw OR (Training, Circuit):ti,ab,kw OR (circuit-type training):ti,ab,kw OR (circuit-type exercise):ti,ab,kw
9	#7 OR #8
10	MeSH descriptor: [Resistance Training] explode all trees
11	(resistance exercise):ti,ab,kw OR (resistance exercise training):ti,ab,kw OR (resistance-type exercise):ti,ab,kw OR (resistance-type training):ti,ab,kw OR (strength training):ti,ab,kw OR (strength-type exercise):ti,ab,kw OR (strength-type training):ti,ab,kw OR (Training, Resistance):ti,ab,kw OR (Training, Strength):ti,ab,kw OR (strength exercise):ti,ab,kw
12	#10 OR #11
13	MeSH descriptor: [Tai Ji] explode all trees
14	(Tai Chi Chuan):ti,ab,kw OR (Tai Ji):ti,ab,kw OR (Chi, Tai):ti,ab,kw OR (Tai Ji Quan):ti,ab,kw OR (Ji Quan, Tai):ti,ab,kw OR (Quan, Tai Ji):ti,ab,kw OR (Taiji):ti,ab,kw OR (Taijiquan):ti,ab,kw OR (Tai-ji):ti,ab,kw OR (T'ai Chi):ti,ab,kw
15	#13 OR #14
16	MeSH descriptor: [Sports] explode all trees
17	(sport):ti,ab,kw OR (Athletics):ti,ab,kw OR (Athletic):ti,ab,kw
18	#16 OR #17
19	MeSH descriptor: [Physical Fitness] explode all trees
20	(fitness):ti,ab,kw OR (fitness, physical):ti,ab,kw OR (cardiorespiratory fitness):ti,ab,kw OR (Fitness, Cardiorespiratory):ti,ab,kw
21	#19 OR #20
22	MeSH descriptor: [Endurance Training] explode all trees
23	(endurance exercise):ti,ab,kw OR (endurance exercise training):ti,ab,kw OR (Training, Endurance):ti,ab,kw

24	#22 OR #23
25	MeSH descriptor: [Walking] explode all trees
26	MeSH descriptor: [Jogging] explode all trees
27	MeSH descriptor: [Running] explode all trees
28	MeSH descriptor: [Bicycling] explode all trees
29	MeSH descriptor: [Swimming] explode all trees
30	MeSH descriptor: [Yoga] explode all trees
31	#25 OR #26 OR #27 OR #28 OR #29 OR #30
32	MeSH descriptor: [Muscle Stretching Exercises] explode all trees
33	(stretching exercise):ti,ab,kw OR (muscle stretching exercises):ti,ab,kw OR (Exercise, Muscle Stretching):ti,ab,kw OR (stretching exercises):ti,ab,kw OR (Muscle Stretching Exercise):ti,ab,kw OR (Static Stretching):ti,ab,kw OR (Stretching, Static):ti,ab,kw OR (Active Stretching):ti,ab,kw OR (Stretching, Active):ti,ab,kw OR (Static-Active Stretching):ti,ab,kw OR (Static Active Stretching):ti,ab,kw OR (Stretching, Static-Active):ti,ab,kw OR (Isometric Stretching):ti,ab,kw OR (Stretching, Isometric):ti,ab,kw OR (Ballistic Stretching):ti,ab,kw OR (Stretching, Ballistic):ti,ab,kw OR (Dynamic Stretching):ti,ab,kw OR (Stretching, Dynamic):ti,ab,kw OR (Proprioceptive Neuromuscular Facilitation (PNF) Stretching):ti,ab,kw OR (PNF Stretching):ti,ab,kw OR (PNF Stretchings):ti,ab,kw OR (Stretching, PNF):ti,ab,kw OR (PNF Stretching Exercise):ti,ab,kw OR (Exercise, PNF Stretching):ti,ab,kw OR (PNF Stretching Exercises):ti,ab,kw OR (Stretching Exercise, PNF):ti,ab,kw OR (Proprioceptive Neuromuscular Facilitation):ti,ab,kw OR (Neuromuscular Facilitation, Proprioceptive):ti,ab,kw OR (Proprioceptive Neuromuscular Facilitations):ti,ab,kw OR (Passive Stretching):ti,ab,kw OR (Stretching, Passive):ti,ab,kw OR (Relaxed Stretching):ti,ab,kw OR (Stretching, Relaxed):ti,ab,kw OR (Static-Passive Stretching):ti,ab,kw OR (Static Passive Stretching):ti,ab,kw OR (Stretching, Static-Passive):ti,ab,kw
34	#32 OR #33
35	MeSH descriptor: [Plyometric Exercise] explode all trees
36	(Exercise, Plyometric):ti,ab,kw OR (Exercises, Plyometric):ti,ab,kw OR (Plyometric Exercises):ti,ab,kw OR (Plyometric Drill):ti,ab,kw OR (Drill, Plyometric):ti,ab,kw OR (Drills, Plyometric):ti,ab,kw OR (Plyometric Drills):ti,ab,kw OR (Plyometric Training):ti,ab,kw OR (Plyometric Trainings):ti,ab,kw OR (Training, Plyometric):ti,ab,kw OR (Trainings, Plyometric):ti,ab,kw OR (Stretch-Shortening Exercise):ti,ab,kw OR (Exercise, Stretch-Shortening):ti,ab,kw OR (Exercises, Stretch-Shortening):ti,ab,kw OR (Stretch Shortening Exercise):ti,ab,kw OR (Stretch-Shortening Exercises):ti,ab,kw OR (Stretch-Shortening Cycle Exercise):ti,ab,kw OR (Cycle Exercise, Stretch-Shortening):ti,ab,kw OR (Cycle Exercises, Stretch-Shortening):ti,ab,kw OR (Exercise, Stretch-Shortening Cycle):ti,ab,kw OR (Exercises, Stretch-Shortening Cycle):ti,ab,kw OR (Stretch Shortening Cycle Exercise):ti,ab,kw OR (Stretch-Shortening Cycle Exercises):ti,ab,kw OR (Stretch-Shortening Drill):ti,ab,kw OR (Drill, Stretch-Shortening):ti,ab,kw OR (Drills, Stretch-Shortening):ti,ab,kw OR (Stretch Shortening Drill):ti,ab,kw OR (Stretch-Shortening Drills):ti,ab,kw
37	#35 OR #36
38	MeSH descriptor: [Aged] explode all trees
39	(senium):ti,ab,kw OR (older adults):ti,ab,kw OR (elder):ti,ab,kw OR (senior):ti,ab,kw OR (older):ti,ab,kw OR (aging):ti,ab,kw OR (elderly):ti,ab,kw
40	#38 OR #39
41	MeSH descriptor: [Aged, 80 and over] explode all trees
42	(Nonagenarians):ti,ab,kw OR (Nonagenarian):ti,ab,kw OR (Oldest Old):ti,ab,kw OR (Centenarians):ti,ab,kw OR (Centenarian):ti,ab,kw OR (Octogenarian):ti,ab,kw OR (Octogenarians):ti,ab,kw OR (very elderly):ti,ab,kw OR (very old):ti,ab,kw
43	#41 OR #42
44	#40 OR #43

45	MeSH descriptor: [Cognitive Dysfunction] explode all trees
46	(mild cognitive impairment):ti,ab,kw OR (Cognitive Dysfunctions):ti,ab,kw OR (Dysfunction, Cognitive):ti,ab,kw OR (Dysfunctions, Cognitive):ti,ab,kw OR (Cognitive Impairments):ti,ab,kw OR (Cognitive Impairment):ti,ab,kw OR (Impairment, Cognitive):ti,ab,kw OR (Impairments, Cognitive):ti,ab,kw OR (Cognitive Impairment, Mild):ti,ab,kw OR (Cognitive Impairments, Mild):ti,ab,kw OR (Impairment, Mild Cognitive):ti,ab,kw OR (Impairments, Mild Cognitive):ti,ab,kw OR (Mild Cognitive Impairments):ti,ab,kw OR (Mild Neurocognitive Disorder):ti,ab,kw OR (Disorder, Mild Neurocognitive):ti,ab,kw OR (Disorders, Mild Neurocognitive):ti,ab,kw OR (Mild Neurocognitive Disorders):ti,ab,kw OR (Neurocognitive Disorder, Mild):ti,ab,kw OR (Neurocognitive Disorders, Mild):ti,ab,kw OR (Cognitive Decline):ti,ab,kw OR (Cognitive Declines):ti,ab,kw OR (Decline, Cognitive):ti,ab,kw OR (Declines, Cognitive):ti,ab,kw OR (Mental Deterioration):ti,ab,kw OR (Deterioration, Mental):ti,ab,kw OR (Deteriorations, Mental):ti,ab,kw OR (Mental Deteriorations):ti,ab,kw
47	#45 OR #46
48	#3 OR #6 OR #9 OR #12 OR #15 OR #18 OR #21 OR #24 OR #31 OR #34 OR #37
49	#48 AND #44 AND #47

Sub-classification of exercise training

Classification	Definition	Illustration
Aerobic exercise	Periodic, dynamic activities involved by the major muscle groups of the whole body	Walking, jogging, running, bicycling, swimming, treadmill, square dancing, aerobics, etc.
Resistance exercise	Any form of exercise that causes the muscles to contract against external resistance with the expectation of increasing muscular strength, mass, and bone density [1]	Exercise by body weight, dumbbell, barbell, elastic band, elastic tube, or other exercise equipment, etc.
Mind body exercise	A sequence of movements and postures with musculoskeletal stretching and relaxation, breath control, and mental focus, have gradually gained global popularity [2]	Yoga, qigong, and tai chi, etc.

[1] Hashida R, Kawaguchi T, Bekki M, Omoto M, Matsuse H, Nago T, Takano Y, Ueno T, Koga H, George J, Shiba Nand Torimura T (2017) Aerobic vs. resistance exercise in non-alcoholic fatty liver disease: A systematic review. *J Hepatol* **66**, 142-152.

[2] Takemura N, Cheung D S T, Smith R, Deng W, Ho K Y, Lin J, Kwok J Y Y, Lam T Cand Lin C C (2020) Effectiveness of aerobic exercise and mind-body exercise in cancer patients with poor sleep quality: A systematic review and meta-analysis of randomized controlled trials. *Sleep Med Rev* **53**, 101334.

Supplemental Outcomes

Study or Subgroup	Aerobic Mean	Aerobic SD	Aerobic Total	Control Mean	Control SD	Control Total	Weight	Std. Mean Difference IV, Random, 95% CI
**1.1.1 The Verbal Learning and Memory Test, direct recall**								
Scherder EJ 2005	41	15.14	15	33.33	16.15	15	22.8%	0.48 [-0.25, 1.20]
**Subtotal (95% CI)**			**15**			**15**	**22.8%**	**0.48 [-0.25, 1.20]**
Heterogeneity: Not applicable								
Test for overall effect: Z = 1.28 (P = 0.20)								
**1.1.2 Chinese Version Verbal Learning Test, total free recall**								
Law LLF 2019	13.69	5.52	16	16.34	4.85	14	22.6%	-0.49 [-1.22, 0.24]
**Subtotal (95% CI)**			**16**			**14**	**22.6%**	**-0.49 [-1.22, 0.24]**
Heterogeneity: Not applicable								
Test for overall effect: Z = 1.33 (P = 0.18)								
**1.1.3 The Rey Auditory Verbal Learning Test**								
Nagamatsu LS 2013	43.38	10.95	24	43	9.61	25	33.4%	0.04 [-0.52, 0.60]
Yogev-Seligmann G 2021	10	2.31	13	10.9	3.41	14	21.2%	-0.30 [-1.06, 0.46]
**Subtotal (95% CI)**			**37**			**39**	**54.6%**	**-0.08 [-0.53, 0.37]**
Heterogeneity: $Tau^2 = 0.00$; $Chi^2 = 0.48$, df = 1 (P = 0.49); $I^2 = 0\%$								
Test for overall effect: Z = 0.35 (P = 0.72)								
**Total (95% CI)**			**68**			**68**	**100.0%**	**-0.05 [-0.45, 0.34]**
Heterogeneity: $Tau^2 = 0.04$; $Chi^2 = 3.94$, df = 3 (P = 0.27); $I^2 = 24\%$								
Test for overall effect: Z = 0.27 (P = 0.79)								
Test for subgroup differences: $Chi^2 = 3.46$, df = 2 (P = 0.18), $I^2 = 42.1\%$								

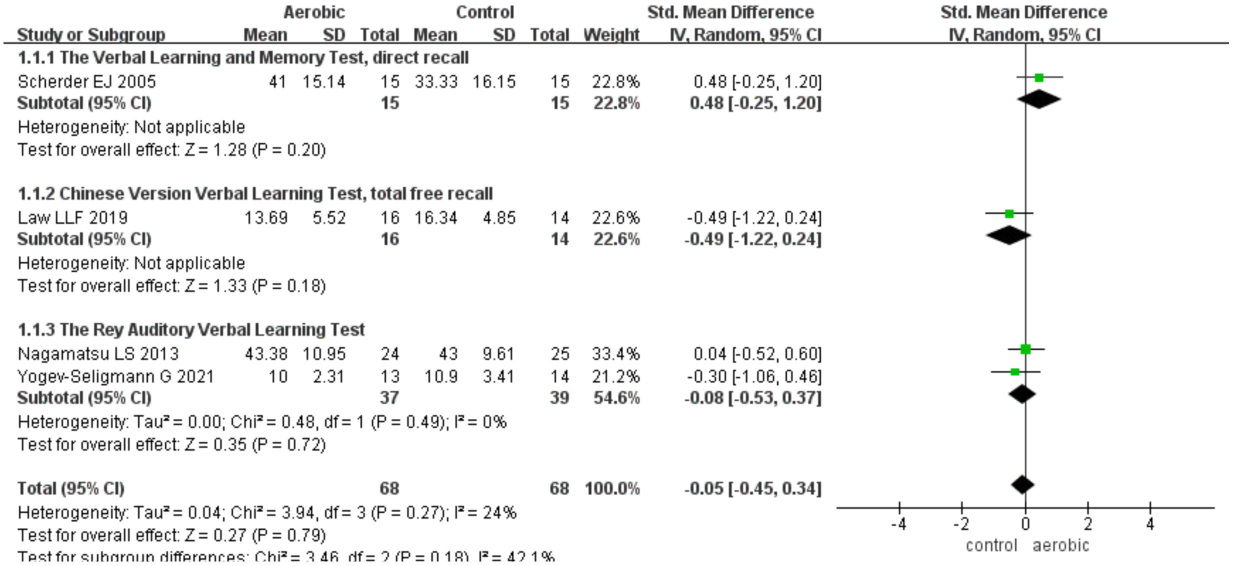

Outcome 1.1 Impact of aerobic exercise training on immediate memory. CI, confidence interval; IV, inverse variance; SD standard deviation; Std, standardized.

Study or Subgroup	Aerobic Mean	Aerobic SD	Aerobic Total	Control Mean	Control SD	Control Total	Weight	Std. Mean Difference IV, Random, 95% CI
**1.2.2 Digit span backward**								
Combourieu Donnezan L 2018	4.72	1.18	18	3.79	0.97	14	24.3%	0.83 [0.10, 1.56]
**Subtotal (95% CI)**			**18**			**14**	**24.3%**	**0.83 [0.10, 1.56]**
Heterogeneity: Not applicable								
Test for overall effect: Z = 2.22 (P = 0.03)								
**1.2.3 Digit span component of the Wechsler memory test**								
Scherder EJ 2005	10.8	3.1	15	10.93	2.69	15	24.9%	-0.04 [-0.76, 0.67]
Tsai CL 2019	19.32	1.77	19	18.78	2.24	18	27.6%	0.26 [-0.39, 0.91]
Yogev-Seligmann G 2021	13.9	4.07	13	15.4	3.37	14	23.2%	-0.39 [-1.15, 0.37]
**Subtotal (95% CI)**			**47**			**47**	**75.7%**	**-0.02 [-0.43, 0.39]**
Heterogeneity: $Tau^2 = 0.00$; $Chi^2 = 1.64$, df = 2 (P = 0.44); $I^2 = 0\%$								
Test for overall effect: Z = 0.10 (P = 0.92)								
**Total (95% CI)**			**65**			**61**	**100.0%**	**0.17 [-0.31, 0.66]**
Heterogeneity: $Tau^2 = 0.11$; $Chi^2 = 5.61$, df = 3 (P = 0.13); $I^2 = 47\%$								
Test for overall effect: Z = 0.69 (P = 0.49)								
Test for subgroup differences: $Chi^2 = 3.97$, df = 1 (P = 0.05), $I^2 = 74.8\%$								

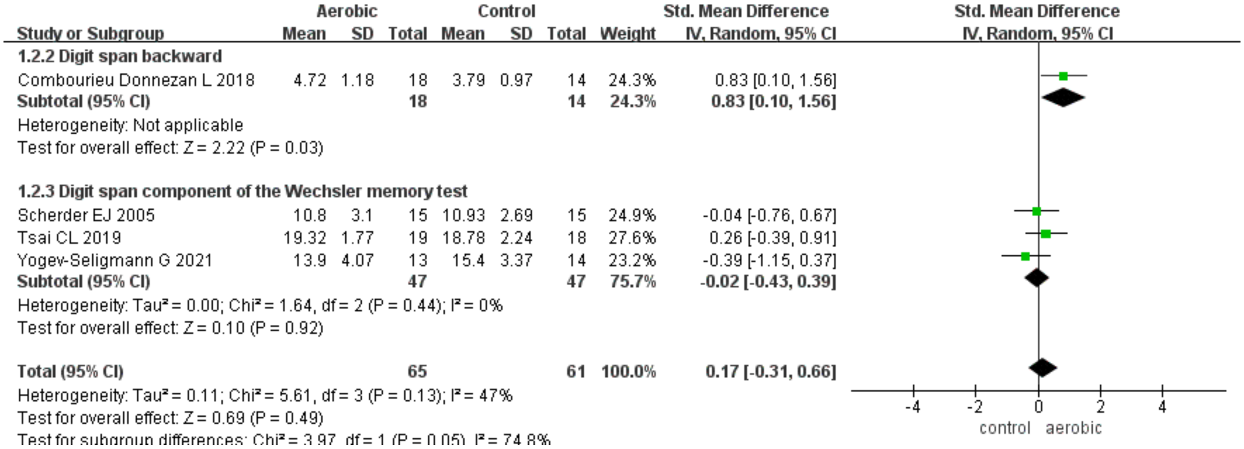

Outcome 1.2 Impact of aerobic exercise training on working memory. CI, confidence interval; IV, inverse variance; SD standard deviation; Std, standardized.

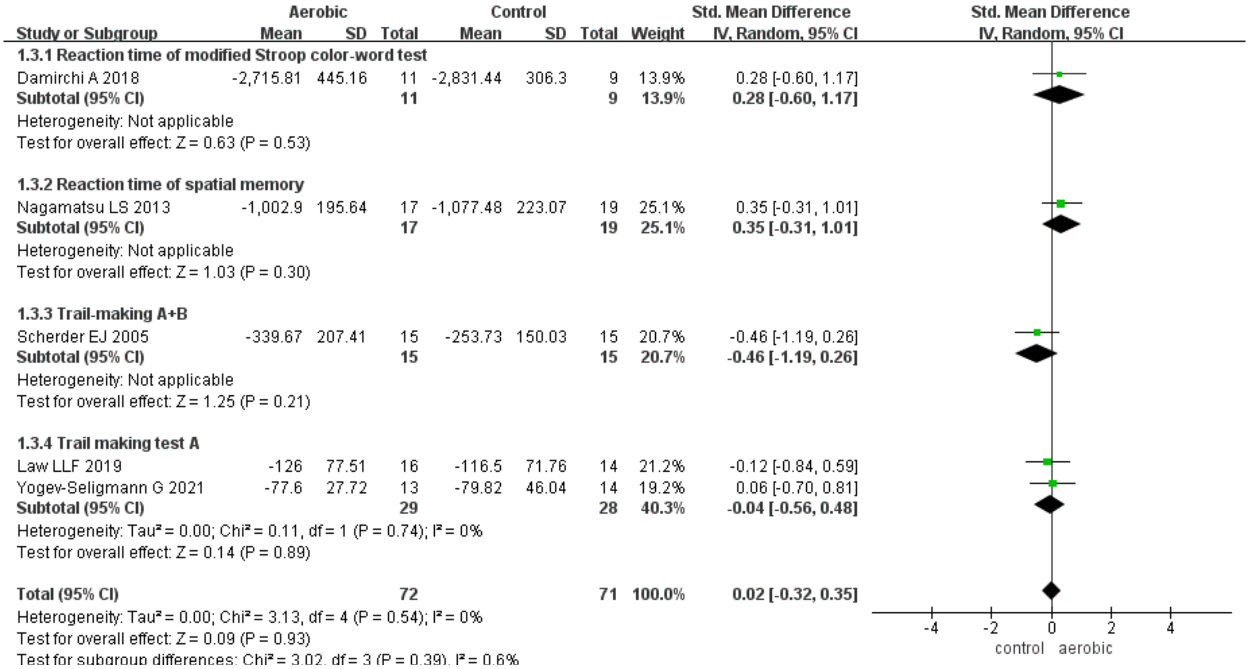

Outcome 1.3 Impact of aerobic exercise training on processing speed. CI, confidence interval; IV, inverse variance; SD standard deviation; Std, standardized.

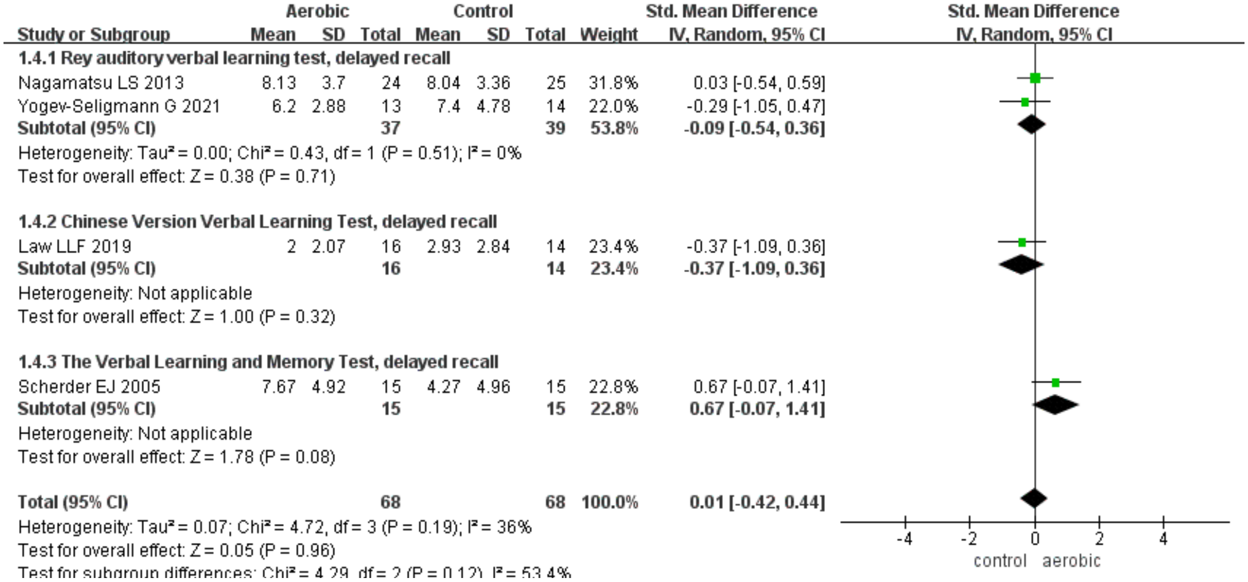

Outcome 1.4 Impact of aerobic exercise training on delayed memory. CI, confidence interval; IV, inverse variance; SD standard deviation; Std, standardized.

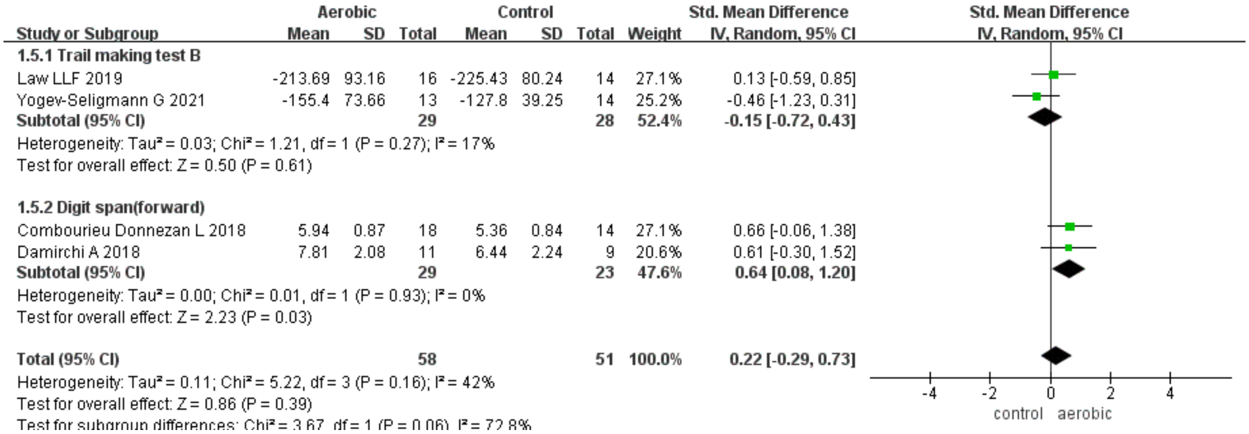

Study or Subgroup	Aerobic Mean	Aerobic SD	Aerobic Total	Control Mean	Control SD	Control Total	Weight	Std. Mean Difference IV, Random, 95% CI
**1.5.1 Trail making test B**								
Law LLF 2019	-213.69	93.16	16	-225.43	80.24	14	27.1%	0.13 [-0.59, 0.85]
Yogev-Seligmann G 2021	-155.4	73.66	13	-127.8	39.25	14	25.2%	-0.46 [-1.23, 0.31]
**Subtotal (95% CI)**			**29**			**28**	**52.4%**	**-0.15 [-0.72, 0.43]**
Heterogeneity: Tau² = 0.03; Chi² = 1.21, df = 1 (P = 0.27); I² = 17%								
Test for overall effect: Z = 0.50 (P = 0.61)								
**1.5.2 Digit span(forward)**								
Combourieu Donnezan L 2018	5.94	0.87	18	5.36	0.84	14	27.1%	0.66 [-0.06, 1.38]
Damirchi A 2018	7.81	2.08	11	6.44	2.24	9	20.6%	0.61 [-0.30, 1.52]
**Subtotal (95% CI)**			**29**			**23**	**47.6%**	**0.64 [0.08, 1.20]**
Heterogeneity: Tau² = 0.00; Chi² = 0.01, df = 1 (P = 0.93); I² = 0%								
Test for overall effect: Z = 2.23 (P = 0.03)								
**Total (95% CI)**			**58**			**51**	**100.0%**	**0.22 [-0.29, 0.73]**
Heterogeneity: Tau² = 0.11; Chi² = 5.22, df = 3 (P = 0.16); I² = 42%								
Test for overall effect: Z = 0.86 (P = 0.39)								
Test for subgroup differences: Chi² = 3.67, df = 1 (P = 0.06), I² = 72.8%								

Outcome 1.5 Impact of aerobic exercise training on attention. CI, confidence interval; IV, inverse variance; SD standard deviation; Std, standardized.

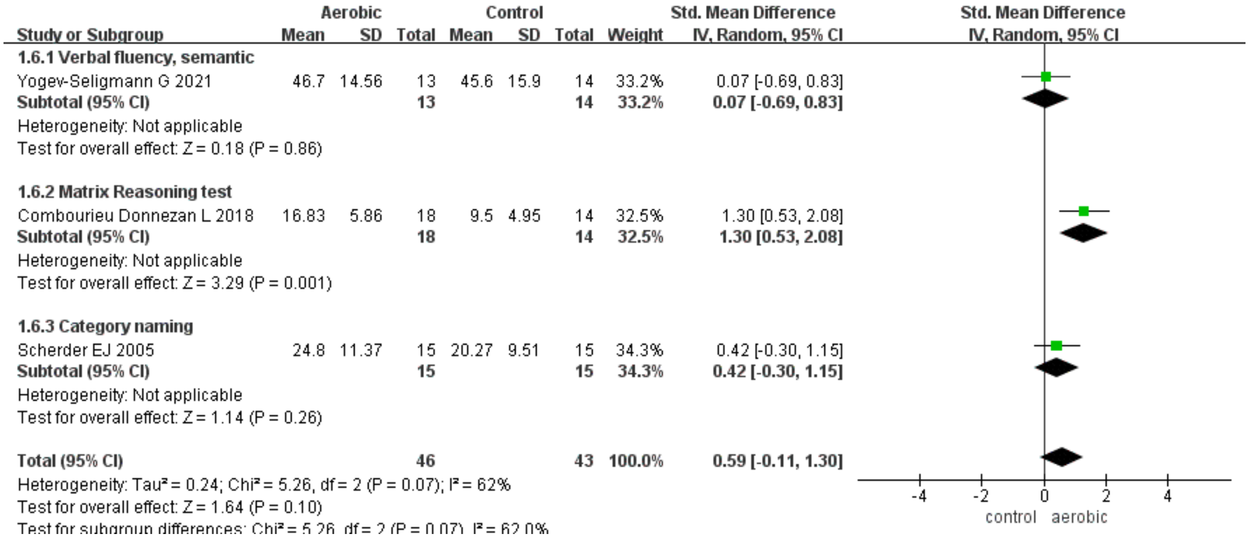

Study or Subgroup	Aerobic Mean	Aerobic SD	Aerobic Total	Control Mean	Control SD	Control Total	Weight	Std. Mean Difference IV, Random, 95% CI
**1.6.1 Verbal fluency, semantic**								
Yogev-Seligmann G 2021	46.7	14.56	13	45.6	15.9	14	33.2%	0.07 [-0.69, 0.83]
**Subtotal (95% CI)**			**13**			**14**	**33.2%**	**0.07 [-0.69, 0.83]**
Heterogeneity: Not applicable								
Test for overall effect: Z = 0.18 (P = 0.86)								
**1.6.2 Matrix Reasoning test**								
Combourieu Donnezan L 2018	16.83	5.86	18	9.5	4.95	14	32.5%	1.30 [0.53, 2.08]
**Subtotal (95% CI)**			**18**			**14**	**32.5%**	**1.30 [0.53, 2.08]**
Heterogeneity: Not applicable								
Test for overall effect: Z = 3.29 (P = 0.001)								
**1.6.3 Category naming**								
Scherder EJ 2005	24.8	11.37	15	20.27	9.51	15	34.3%	0.42 [-0.30, 1.15]
**Subtotal (95% CI)**			**15**			**15**	**34.3%**	**0.42 [-0.30, 1.15]**
Heterogeneity: Not applicable								
Test for overall effect: Z = 1.14 (P = 0.26)								
**Total (95% CI)**			**46**			**43**	**100.0%**	**0.59 [-0.11, 1.30]**
Heterogeneity: Tau² = 0.24; Chi² = 5.26, df = 2 (P = 0.07); I² = 62%								
Test for overall effect: Z = 1.64 (P = 0.10)								
Test for subgroup differences: Chi² = 5.26, df = 2 (P = 0.07), I² = 62.0%								

Outcome 1.6 Impact of aerobic exercise training on executive function. CI, confidence interval; IV, inverse variance; SD standard deviation; Std, standardized.

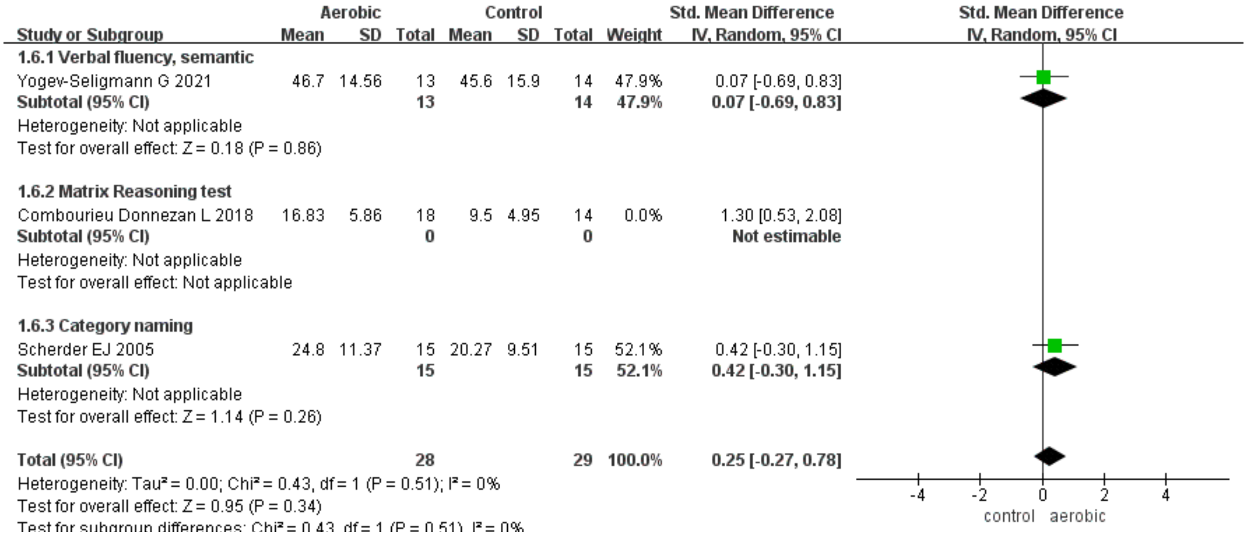

Outcome 1.6.1 Impact of aerobic exercise training on executive function without Combourieu Donnezan L et al. included. CI, confidence interval; IV, inverse variance; SD standard deviation; Std, standardized.

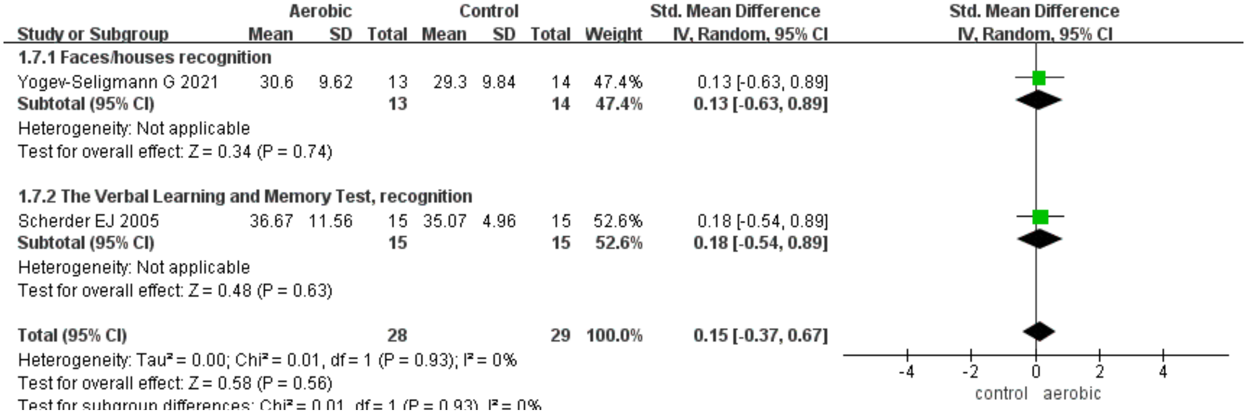

Outcome 1.7 Impact of aerobic exercise training on recognition. CI, confidence interval; IV, inverse variance; SD standard deviation; Std, standardized.

Study or Subgroup	Aerobic Events	Aerobic Total	Control Events	Control Total	Weight	Odds Ratio M-H, Random, 95% CI
Combourieu Donnezan L 2018	3	21	1	15	16.6%	2.33 [0.22, 24.92]
Damirchi A 2018	4	15	0	9	10.0%	7.43 [0.35, 156.28]
Law LLF 2019	2	16	0	14	9.5%	5.00 [0.22, 113.50]
Nagamatsu LS 2013	6	30	1	28	19.4%	6.75 [0.76, 60.15]
Scherder EJ 2005	0	15	0	15		Not estimable
Tsai CL 2019	3	22	4	22	35.0%	0.71 [0.14, 3.63]
Yogev-Seligmann G 2021	2	15	0	14	9.5%	5.37 [0.24, 122.29]
**Total (95% CI)**		**134**		**117**	**100.0%**	**2.47 [0.94, 6.49]**
Total events	20		6			

Heterogeneity: Tau² = 0.00; Chi² = 4.05, df = 5 (P = 0.54); I² = 0%
Test for overall effect: Z = 1.84 (P = 0.07)

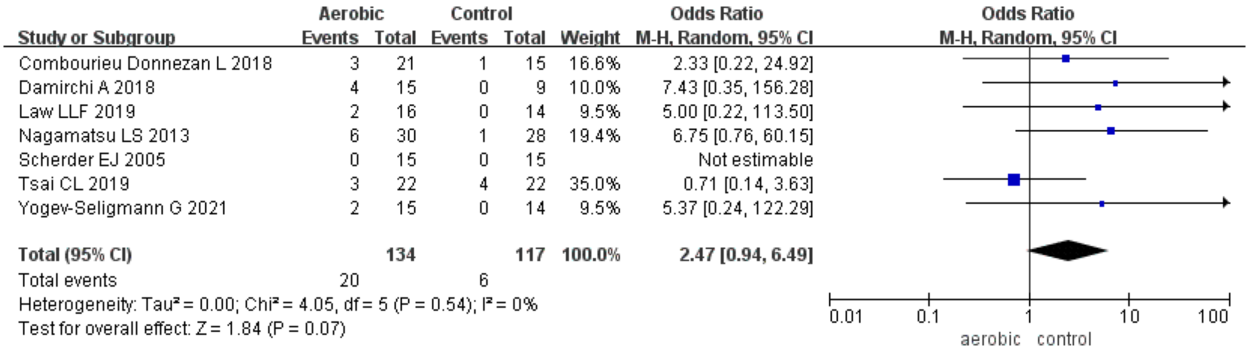

Outcome 1.8 Dropout rates between aerobic exercise groups and control groups. CI, confidence interval; M-H, Mantel-Haenszel.

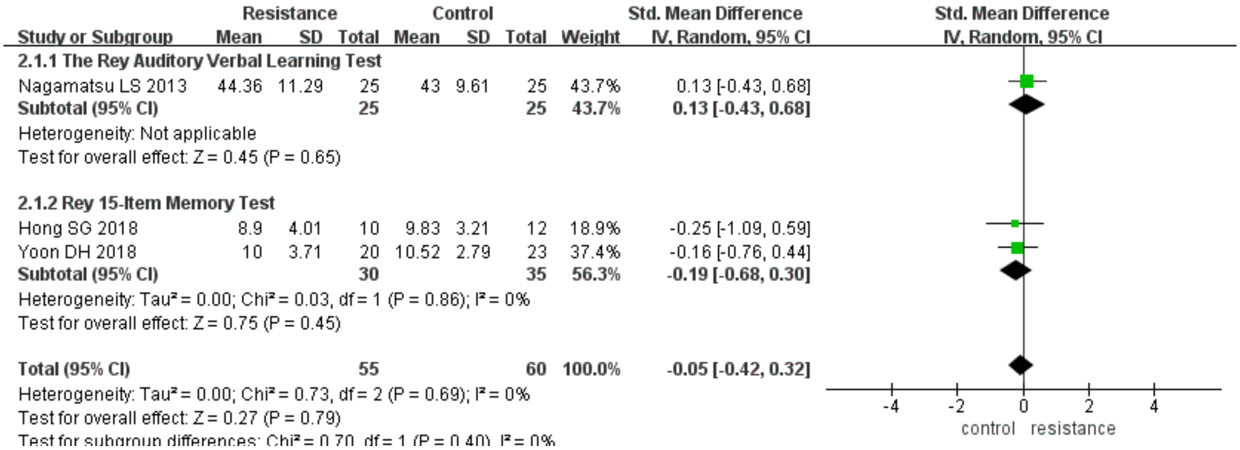

Outcome 2.1 Impact of resistance exercise training on immediate memory. CI, confidence interval; IV, inverse variance; SD standard deviation; Std, standardized.

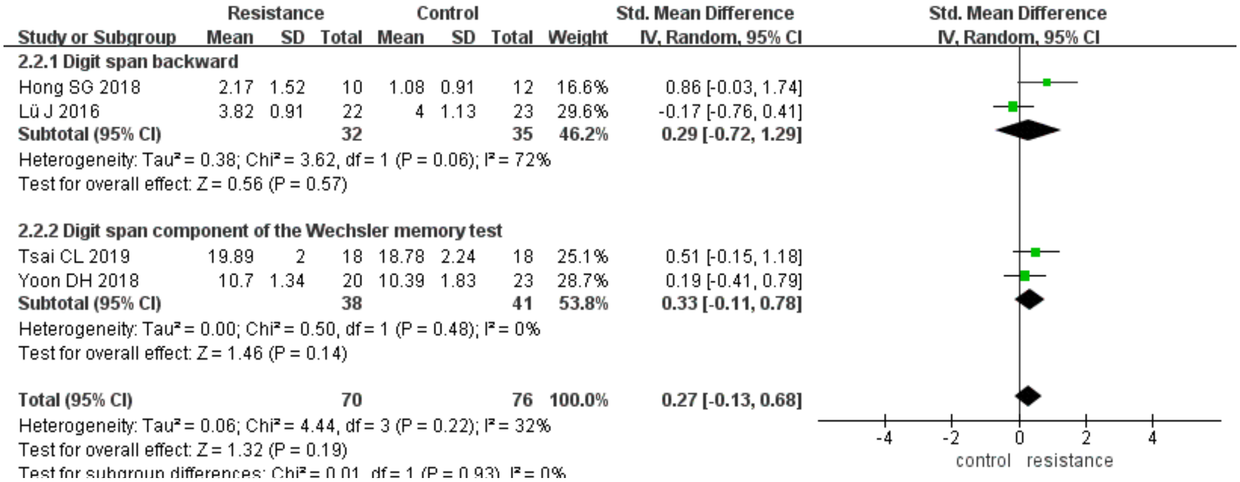

Outcome 2.2 Impact of resistance exercise training on working memory. CI, confidence interval; IV, inverse variance; SD standard deviation; Std, standardized.

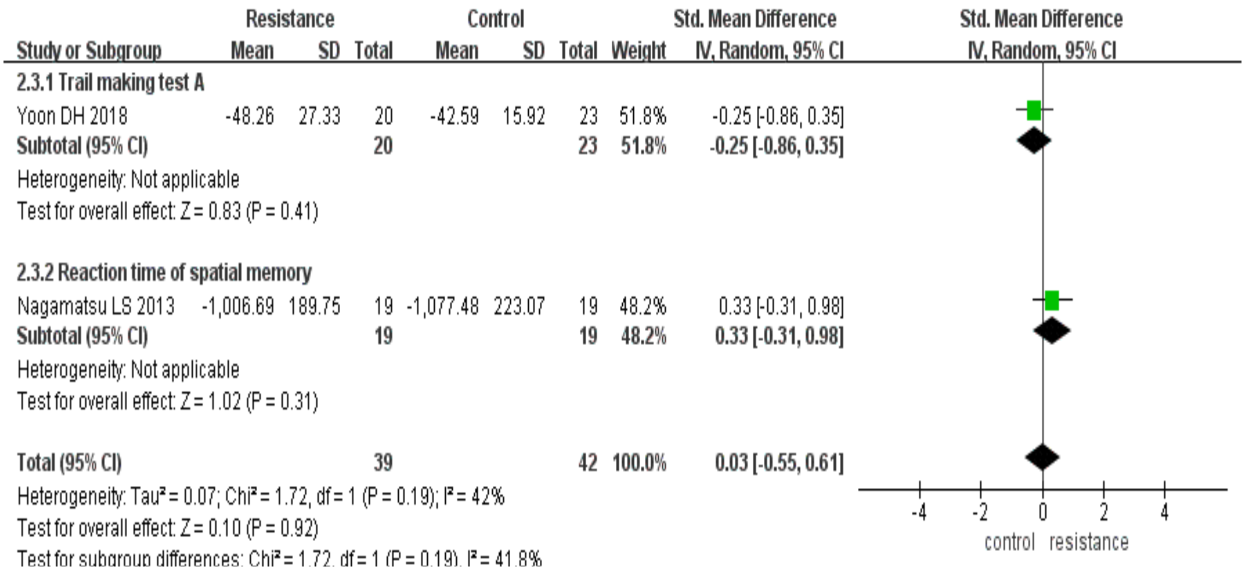

Outcome 2.3 Impact of resistance exercise training on processing speed. CI, confidence interval; IV, inverse variance; SD standard deviation; Std, standardized.

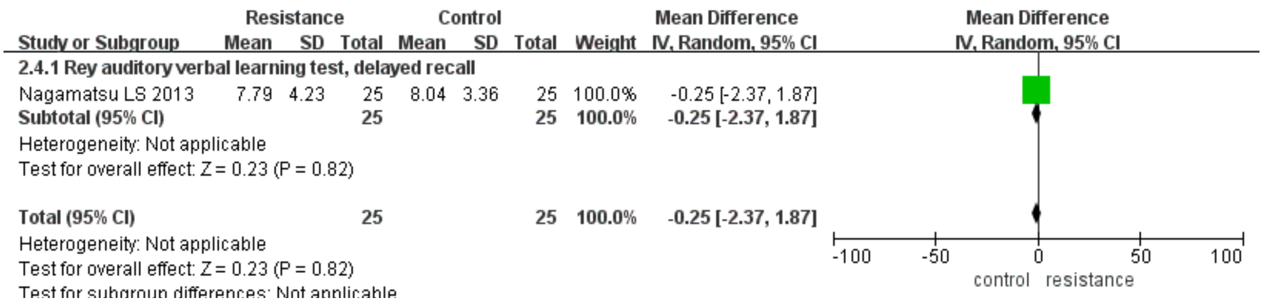

Outcome 2.4 Impact of resistance exercise training on delayed memory. CI, confidence interval; IV, inverse variance; SD standard deviation; Std, standardized.

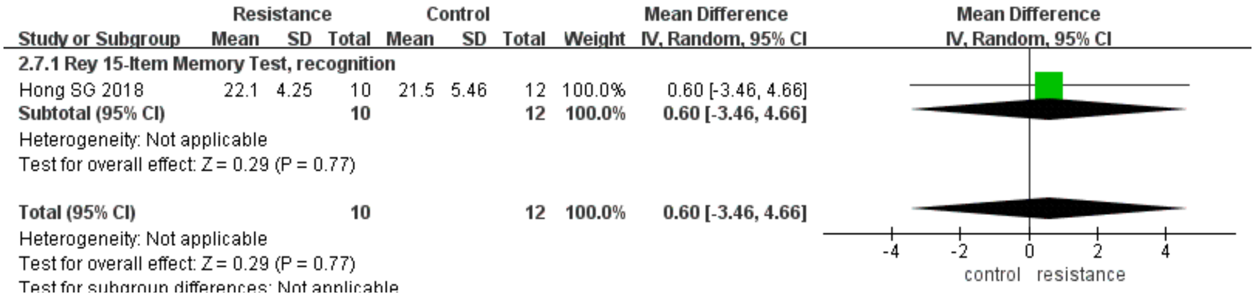

Outcome 2.7 Impact of resistance exercise training on recognition. CI, confidence interval; IV, inverse variance; SD standard deviation; Std, standardized.

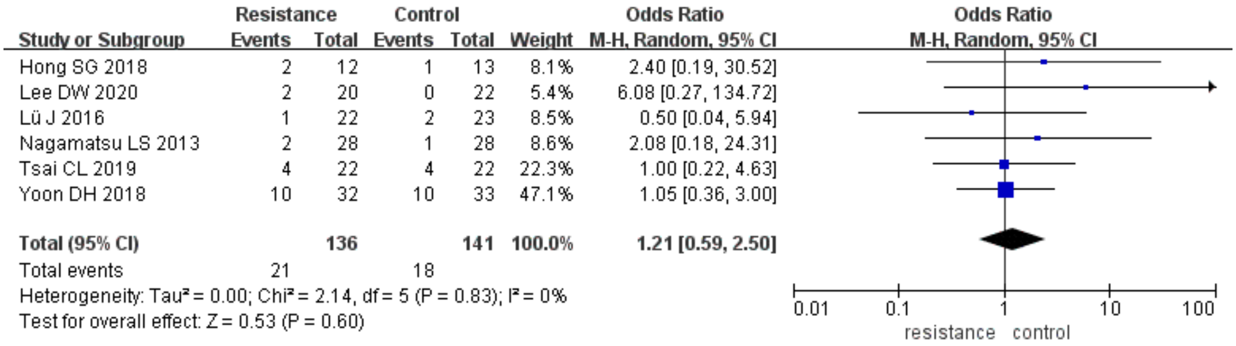

Outcome 2.8 Dropout rates between resistance exercise groups and control groups. CI, confidence interval; M-H, Mantel-Haenszel.

Study or Subgroup	Multimodal Mean	Multimodal SD	Multimodal Total	Control Mean	Control SD	Control Total	Weight	Mean Difference IV, Random, 95% CI
**3.1.1 List learning delayed recall test**								
Lam LC 2015	5.3	2.4	114	5.6	2.2	101	100.0%	-0.30 [-0.91, 0.31]
**Subtotal (95% CI)**			**114**			**101**	**100.0%**	**-0.30 [-0.91, 0.31]**
Heterogeneity: Not applicable								
Test for overall effect: Z = 0.96 (P = 0.34)								
**Total (95% CI)**			**114**			**101**	**100.0%**	**-0.30 [-0.91, 0.31]**
Heterogeneity: Not applicable								
Test for overall effect: Z = 0.96 (P = 0.34)								
Test for subgroup differences: Not applicable								

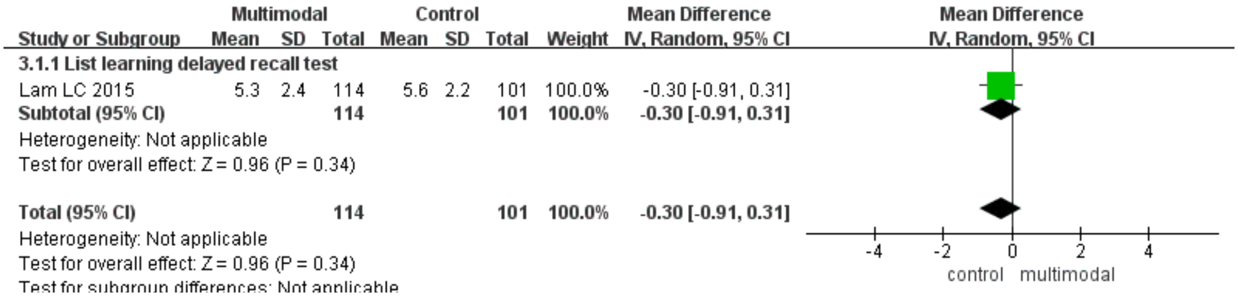

Outcome 3.1 Impact of multimodal exercise training on delayed memory. CI, confidence interval; IV, inverse variance; SD standard deviation; Std, standardized.

Study or Subgroup	Multimodal Mean	Multimodal SD	Multimodal Total	Control Mean	Control SD	Control Total	Weight	Mean Difference IV, Random, 95% CI
**3.2.1 Category verbal fluency test**								
Lam LC 2015	34.3	8	114	35.5	7.8	101	100.0%	-1.20 [-3.31, 0.91]
**Subtotal (95% CI)**			**114**			**101**	**100.0%**	**-1.20 [-3.31, 0.91]**
Heterogeneity: Not applicable								
Test for overall effect: Z = 1.11 (P = 0.27)								
**Total (95% CI)**			**114**			**101**	**100.0%**	**-1.20 [-3.31, 0.91]**
Heterogeneity: Not applicable								
Test for overall effect: Z = 1.11 (P = 0.27)								
Test for subgroup differences: Not applicable								

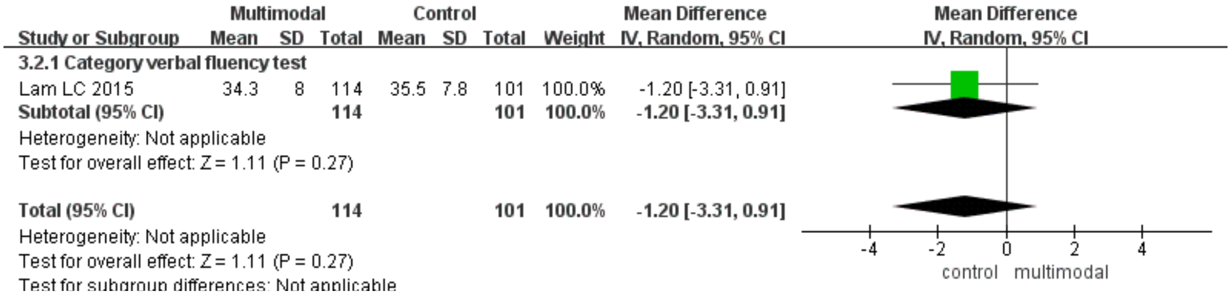

Outcome 3.2 Impact of multimodal exercise training on executive function. CI, confidence interval; IV, inverse variance; SD standard deviation; Std, standardized.

Study or Subgroup	Multimodal Events	Multimodal Total	Control Events	Control Total	Weight	Odds Ratio M-H, Random, 95% CI
Lam LC 2015	33	147	30	131	100.0%	0.97 [0.56, 1.71]
**Total (95% CI)**		**147**		**131**	**100.0%**	**0.97 [0.56, 1.71]**
Total events	33		30			
Heterogeneity: Not applicable						
Test for overall effect: Z = 0.09 (P = 0.93)						

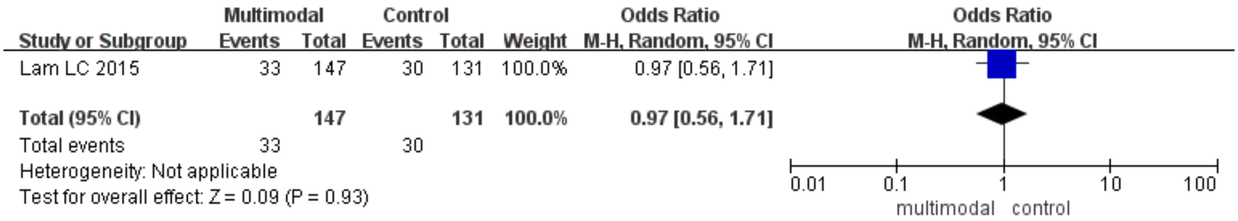

Outcome 3.3 Dropout rates between multimodal exercise groups and control groups. CI, confidence interval; M-H, Mantel-Haenszel.
